# Constituents of *Chamaecrista diphylla* (L.) Greene Leaves with Potent Antioxidant Capacity: A Feature-Based Molecular Network Dereplication Approach

**DOI:** 10.3390/pharmaceutics13050681

**Published:** 2021-05-10

**Authors:** Paulo Gomes, Luis Quirós-Guerrero, Abraão Muribeca, José Reis, Sônia Pamplona, Anderson H. Lima, Mariele Trindade, Consuelo Silva, Jesus N. S. Souza, Jean Boutin, Jean-Luc Wolfender, Milton Silva

**Affiliations:** 1 Laboratory of Liquid Chromatography (Labcrol), Institute of Exact and Natural Sciences, Federal University of Pará, Belém 66075-110, Brazil; wendergomes@ufpa.br (P.G.); abraao_muribeca@hotmail.com (A.M.); reisdiogo190@gmail.com (J.R.); sgpamplona@yahoo.com.br (S.P.); yumikoyoshioka@yahoo.com.br (C.S.); 2 School of Pharmaceutical Sciences, University de Geneva, CMU, Rue Michel-Servet 1, CH-1206 Geneva, Switzerland; Luis.Guerrero@unige.ch (L.Q.-G.); Jean-Luc.Wolfender@unige.ch (J.-L.W.); 3 Institute of Pharmaceutical Sciences of Western Switzerland, University of Geneva, CMU, Rue Michel Servet 1, 1211 Geneva, Switzerland; 4 Drug Planning and Development Laboratory, Federal University of Pará, Belém 66075-110, Brazil; anderson.smg@gmail.com; 5 Faculty of Food Engineering, Federal University of Pará, Belém 66075-110, Brazil; mariectrindade@gmail.com (M.T.); jsouza@ufpa.br (J.N.S.S.); 6 Faculty of Pharmaceutical Sciences, Federal University of Pará, Belém 66075-110, Brazil; 7 Center for the Valorization of Bioactive Compounds in the Amazon (CVACBA), Federal University of Pará, Belém 66075-110, Brazil; 8 PHARMADEV (Pharmacochimie et Biologie pour le Développement), Université Toulouse 3 Paul Sabatier, Faculté de Pharmacie, CEDEX 9, 31062 Toulouse, France; ja.boutin.pro@gmail.com

**Keywords:** *Chamaecrista diphylla*, antioxidants, dereplication, molecular networking, bioactive molecules

## Abstract

*Chamaecrista diphylla* (L.) Greene (Fabaceae/Caesalpiniaceae) is a herbaceous plant that is widely distributed throughout the Americas. Plants from this genus have been used in traditional medicine as a laxative, to heal wounds, and to treat ulcers, snake and scorpion bites. In the present study, we investigated the chemical composition of *Chamaecrista diphylla* leaves through a mass spectrometry molecular network approach. The oxygen radical absorbance capacity (ORAC) for the ethanolic extract, enriched fractions and isolated compounds was assessed. Overall, thirty-five compounds were annotated for the first time in *C. diphylla*. Thirty-two of them were reported for the first time in the genus. The isolated compounds 9, 12, 24 and 33 showed an excellent antioxidant capacity, superior to the extract and enriched fractions. Bond dissociation energy calculations were performed to explain and sustain the antioxidant capacity found. According to our results, the leaves of *C. diphylla* represent a promising source of potent antioxidant compounds.

## 1. Introduction

Oxidative stress is produced when cells cannot eliminate the excess of reactive oxygen species (ROS) produced by the mitochondria; it is directly related to biological aging [1]. Oxidative stress is a major cofactor for cardiovascular and neurodegenerative diseases, as well as cancer, diabetes and acute inflammatory problems [2]. Every day, humans are exposed to several biotic and abiotic factors, like UV radiation, allergens, toxins or factors arising from an unhealthy lifestyle (poor diet, physical morbidity, etc.), contributing to oxidative stress [3]. The pharmaceutical industry implements natural and synthetic antioxidants, substances that inhibit and or decrease the intracellular levels of reactive oxygen or nitrogen species [4]. Common antioxidants used in the industry include butylated hydroxyanisole (BHA), hydroxytoluene (BHT), propyl gallate (PG) and tert-butylhydroquinone (TBHQ) [5]. However, some studies have shown that some of these compounds are not completely safe, due to their off-target actions, particularly their inhibitory potency towards numerous enzymes [6]. Their substitution is encouraged, preferably with natural antioxidants that show high biodegradability, lower toxicity and safer mechanisms of action [7]. This type of antioxidant molecules is commonly present in red fruits, seeds, wines, teas, onion bulbs, olive oils and aromatic plants [8]. One of the most studied compounds in this line is melatonin [9], although its mechanisms of action are still controversial [10].

Plants from the *Chamaecrista* genus are widely used in traditional medicine in several regions of the Americas, Africa and Asia. They present several therapeutic properties as laxatives (*C. biensis, C. cathartica* and *C. lateriticola*), wound and ulcer treatments (*C. absus* and *C. nigricans*) and show anti-ophidic effects against snake and scorpion poisons (*C. apoucouita*) [11,12]. Most medicinal plants are prepared as infusions or unguents [13]. *Chamaecrista diphylla* (L.) Greene (Fabaceae/Caesalpiniaceae) is widely distributed in the savannas and coastal areas of Mexico, Central America (Belize, Cuba, Guatemala, Caribbean Islands, Panama and Puerto Rico) and South America (Brazil, Colombia, Guyana and Venezuela) [14,15]. Described as a small herb, this plant grows up to 15 cm and develops branches from a single point fixed to the ground. In Brazil, is commonly known as “manduberana”, “mandubirana”, “mendubi” and “senna do campo” [16]. The phytochemistry of this plant has not been exploited or described extensively in literature. Most of the works are centered in the phylogeny and taxonomical classification of plants from the genus. The few reports found indicate an interesting antioxidant activity for this plant, caused apparently by a high content of flavonoids and phenolics [17].

Our group has been involved in the description of the secondary metabolite compositions of several Amazonian plants [18,19,20], a starting point for finding renewal and sustainable sources of natural compounds [21] and an inspiring alternative to medicinal chemistry programs [22]. Based on previous studies, it is conceivable that *Chamaecrista diphylla* has an underlying value which can promote its use in alimentary, pharmaceutical and cosmetic applications. The present study reports the antioxidant capacity, total phenolic content, total flavonoid content and the putative identity of a great number of metabolites present in *Chamaecrista diphylla* leaves.

## 2. Materials and Methods

### 2.1. Plant Material

Leaves of *Chamaecrista diphylla* (L.) Greene were collected from mature plants with flowers in the coastal strip of the Bacurizal Ecological Reserve, located under geographical coordinates W 048°31′07.0 and S 00°46′41.0, municipality of Salvaterra, Pará, Brazil. The collected material was identified and deposited in the herbarium of Pará State University by Professor Dr Marlene Freitas da Silva (voucher 008383). Permission to access the Brazilian genetic patrimony was provided by SISGEN (AAA68AD).

The leaves of *C. diphylla* were washed with ultrapure water from a Direct-Q 5 system (Millipore, Darmstadt, Germany) and sprayed with 70% ethanol (*w*/*w*, Minas Gerais, Brazil). Samples were dried in an air circulation oven (Quimis, Brazil) at 45 °C until reaching a constant weight. The dry material was crushed in a ball mill until reaching 60–100 µm granulometry.

### 2.2. Extraction and Sample Preparation

The dry crushed leaves (116 g) were macerated with 500 mL of ethanol (Tedia, Fairfield, OH, USA) twice with 24 h agitation each time. Both extract volumes were combined and concentrated under a vacuum in a rotary evaporator at 45 °C (Büchi, Flawil, Germany). The residue was dried in an air circulation oven at 45 °C until reaching a constant weight, resulting in 48.0 g of crude extract (41.37% yield).

Then, 20 g of extract were fractionated on a silica gel 60–200 µm (Sigma-Aldrich, Saint Louis, MO, USA) column (4.8 × 70 cm). First, 400 mL of hexane/ethyl acetate 1:1 were passed through the extract, in order to remove less polar nuisance compounds, and discarded. Then, 400 mL of ethyl acetate (Fr-OAcET) and 400 mL methanol (Fr-MeOH) were used. The two fractions were collected separately and concentrated to dryness at 38 °C in a rotatory evaporator until reaching a constant weight.

The samples (crude extract and fractions) were subjected to prefiltration with solid phase extraction (SPE) C_18_ cartridges of 100 mg (Phenomenex, Torrance, CA, USA) to remove interfering compounds before carrying out any tests. The cartridges were previously conditioned with 1 mL of methanol and 1 mL of ultra-pure water. Then, 10 mg of sample was dissolved in 1 mL of MeOH:H_2_O (80:20 *v*/*v*) and filtered through the cartridge. The solvent was collected in a vial. Then, 1 mL of the same solvent mixture was passed through the cartridge and collected in the same vial. The solvent was dried and the residue was weighed. The resulting sample was used for antioxidant and UHPLC-MS/MS experiments.

### 2.3. Isolation of Compounds

The Fr-OAcEt (736.36 mg) was dissolved in 4.8 mL of methanol. A volume of 1.2 mL of H_2_O was added and mixed under sonication for 1 min. The solution was passed through a solid phase extraction C_18_ cartridge (SPE, Phenomenex, 1000 mg). After evaporation, the residue (101.3 mg) was injected and separated in an HPLC model LC-6AD with a SPD-10AV UV diode detector (Shimadzu, Tokyo, Japan) using a semi-preparative Gemini C_18_ (250 × 10 mm, 5 µm, 110 A) column and an elution system of H_2_O:MeOH:ACN (60:24:16 *v*/*v*) at 4.7 mL mL^−1^ for 40 min. Collection was performed based on the UV trace at 254 nm and 330 nm. This separation yielded compounds 9 (8.9 mg, RT 7.55 min), 12 (9.7 mg, RT 10.09 min), 17 (8.7 mg, RT 20.42 min), 20 (5 mg, RT 24.86 min), 24 (6.8 mg, RT 29.06 min), 26 (5.7 mg, RT 30.04 min), 29 (6.7 mg, RT 34.29 min) and 33 (5.2 mg, RT 37.18 min).

### 2.4. Determination of the Total Phenols

The total phenolic content was determined using the Folin–Ciocalteu reagent method as described by [23] for both the extract and the fractions. The total phenolic content was expressed as milligrams of gallic acid equivalent per gram of sample (mg GAE/g) using gallic acid as a standard. The absorbance was measured at k = 750 nm in a Kasuaki UV-VIS 1020 nm spectrophotometer (Model IL-592, Wuxi, China). Gallic acid standard curve y = 0.0994x + 0.0108, R^2^ = 0.999.

### 2.5. Determination of Total Flavonoids

The content of flavonoids was determined using the *p*-dimethylaminocinnamaldehyde (DMACA) method described by [24] for both the extract and fractions. The results were expressed in mg equivalent of catechin (mg CE/g sample) using catechin as a standard. Catechin standard curve y = 0.1017x + 0.0497, R^2^ = 0.9956

### 2.6. Scavenging Antioxidant Activity

The antioxidant activity was measured using the oxygen radical absorbance capacity (ORAC) assay, using fluorescein as a fluorescent probe [25,26]. The ORAC experiments were performed on a Synergy HTX spectrophotometer (BIOTEK, USA), using a 96-well microplate [27]. The fluorescence intensity (485 nm/525 nm) was checked every minute for 50 min. The plate reader was controlled using Gen 5 software version 2.07 (BioTek Instruments, Inc., Winooski, VT, USA). All reagents were prepared in sodium phosphate buffer, pH 7.4, and the final reaction volume was 200 µL.

A total of 5 mg of ethanolic extract, Fr-MeOH and Fr-OAcEt (after SPE) were dissolved in 2 mL of methanol (2500 ppm, stock solution). From the stock solution, a second 50-fold dilution was made (solution 2). From solution 2, serial dilutions of 4000, 8000 and 12,000-fold were prepared. The samples were analyzed in these three concentrations (0.0125 µg mL^−1^, 0.00625 µg mL^−1^ and 0.00417 µg mL^−1^) in duplicate. Measurements were done as follows: 25 µL of the sample were mixed with 150 µL of fluorescein solution (0.5 mM) and incubated for 15 min at 37 °C. Subsequently, 25 µL of 2,2′-azobis (2-amidinopropane) dihydrochloride solution (AAPH) 0.15 M were automatically added and stirred. The blank consisted of fluorescein and AAPH with sodium phosphate buffer. Trolox solutions (1, 2, 4 and 8 µM, in triplicates) obtained from Sigma-Aldrich (Darmstadt, Germany) were used as an antioxidant reference to build the quantification curve. ORAC indices were calculated (net area under the curve (AUC)) as indicated in the literature [25,27], expressed in mmol Trolox equivalents/g dry sample (mmol TE/g dry sample). The isolated compounds were measured in the same concentrations as those for the extract and fractions.

### 2.7. Calculation of Bond Dissociation Enthalpies O–H

Bond dissociation enthalpy (BDE) was determined as the difference in the heat of formation between the molecule and its corresponding radical. Firstly, we carried out geometry optimizations at the PM6 [28] level to speed up the convergence. Then, optimizations, vibrational frequencies and energetic analysis of the neutral molecules and their radicals were computed, employing density functional theory at the ωB97XD/6-31G(d) level using Gaussian 09 software, Revision D.01 (Gaussian, Inc., Pittsburgh, PA, USA). Long-range corrected functionals such as ωB97XD [29] benefitted from the inclusion of Grimme’s D2 dispersion model of dispersion effects. The total energy (E) of each species includes electronic energy and zero-point correction (ZPE) generated based on a vibrational frequency calculation. Finally, polarizable continuum model (PCM) calculations were employed to introduce the solvent effect in BDE calculations.

### 2.8. UHPLC-MS/MS Analysis

The samples at 1 mg mL^−1^ in methanol:H_2_O (80:20) were analyzed on a Xevo^®^ G2-S QTof coupled with an ACQUITY Ultra Performance LC™ system (Waters Corp., Milford, MA, USA). The column and autosampler temperatures were set at 40 °C and 25 °C, respectively. Leucine-enkephalin was used as a reference compound. The samples were analyzed in a BEH C18 column (50 × 2.1 mm; 1.7-μm particle size; Waters, Milford, USA) using ultra-pure water with 0.1% formic acid (solvent A) and methanol with 0.1% formic acid (solvent B). The injection volume was 5.0 μL and the flow rate 300 μL/min. A linear gradient elution of 5–60% of B in 20 min was used. The mass spectrometry parameters were set to desolvation gas flow (N_2_) at 600 L/h and desolvation temperature at 150 °C, the cone gas flow (N_2_) at 50 L/h and the source temperature at 120 °C. The capillary and sampling cone voltages were adjusted to 1.0 kV and 40 V, respectively. The detection was performed using different MS functions with a scan time and interscan time of 0.3 s and 0.014 s, respectively. The data-dependent acquisition (DDA) was performed at 20 min, negative ionization range of 50–1200 Da, centroid, top-5 experiment and normalized collision energy (NCE) set to increase from 10 to 50. A scan rate of 0.5 s, charge states of +1 and +2, tolerance +/− of 0.2 Da, peak extraction of 2 Da, tolerance of isotope +/− 3 Da and extraction tolerance of isotope 6 Da were used. MassLynx 4.1 software (Waters, Milford, USA) was used for data acquisition.

### 2.9. Metabolite Characterization Workflow

An in-house database of compounds restricted to the genus *Chamaecrista* (L.) Moench was built, including their spectrometric data. This database contains a total of 117 entries of varied chemical classes, such as flavones, xanthones, anthraquinones, terpenes and fatty acids [30,31,32,33,34].

The execution files of the ethanolic extract (EE), methanolic fraction (Fr-MeOH) and ethyl acetate fraction (Fr-OAcEt) acquired in the Xevo G2-S QTof mass spectrometer were converted to. mzML format using MS Convert [35]. Then, the data were processed in MZmine 2.53 [36]. The minimum intensity level for MS level 1 detection in negative ionization was set at 1.0 × 10^3^ and MS level 2 at 5.0 × 10^1^. Chromatograms were constructed using ADAP (Automated Data Analysis Pipeline) with minimum group size of 2 and intensity limit of 3.0 × 10^3^, highest intensity value of 3.0 × 10^3^ and a *m*/*z* tolerance of 10 ppm. The local minimum search algorithm was used to deconvolute the chromatogram, with *m*/*z* tolerance values of 0.5 and 0.2 min for the pairing of MS/MS and RT, respectively. Isotopes were detected using a tolerance of 10 ppm, an RT tolerance of 0.2 min and the maximum charge of 2. For compound alignment, the tolerance of *m*/*z* 10 ppm was used, and scores for *m*/*z* of 75 and 25 for RT with a tolerance of 0.2 min. The resulting compound list was filtered to remove features not containing associated MS/MS spectra and then subjected to gap-filling. Finally, the corresponding files were exported for feature-based molecular networking (FBMN) in GNPS.

A feature-based molecular network (FBMN) was created using the exported files. Additionally, metadata with attributes to distinguish ethanol extract and fractions were created according to the workflow recommendations available on the GNPS platform [37]. The parameters used for FBMN were as follows—precursor ion mass and fragment tolerance set at 0.02 Da, a cosine score equal to or greater than 0.7, topK set at 10, minimum matched fragment ions set to 6 and maximum node connection equal to 100. The same parameters were used to search the GNPS spectral libraries. The job record is available at https://gnps.ucsd.edu/ProteoSAFe/status.jsp?task=177c35c2f7d44bc3960aaab12613fb02. The output of the GNPS platform was used to annotate against the ISDB-DNP (In Silico DataBase-Dictionary Natural Products) [38] and then the script for taxonomically informed metabolite annotation [39] was used to re-rank and clean the output based on the taxonomy. Visualization and treatment of the molecular network was performed using Cytoscape 3.8.2 software [40].

## 3. Results

### 3.1. Antioxidant Activity Assessment of Chamaecrista diphylla Extract and Enriched Fractions

The ethanolic extract (EE) of *Chamaecrista diphylla* leaves showed an important ORAC activity (4.29 ± 0.20 mmol TE/g), indicating the presence of potent antioxidant compounds. To locate the compounds responsible for this activity, the EE was subjected to silica gel column chromatography to produce methanolic (Fr-MeOH) and ethyl acetate (Fr-OAcEt) enriched fractions. Both presented different chromatographic profiles (Supplementary Materials, Figure S1) and higher ORAC values than the original EE (See Table 1). The major compounds found in the EE chromatographic trace were found in Fr-MeOH, whereas the minor compounds at higher retention times were concentrated in Fr-OAcEt.

Additionally, the total phenolic and total flavonoid contents were determined, using the Folin–Ciocalteu and the *p*-dimethylaminocinnamaldehyde methods, respectively (see Table 1). These results suggest the presence of high amounts of phenolic-like compounds among others in the original extract and enriched fractions, which could be responsible for the observed activity. Specially in Fr-OAcEt, the values were almost twice as great as than those for EE and Fr-MeOH.

### 3.2. Dereplication and Isolation of Compounds from Chamaecrista diphylla

**Figure 1:**
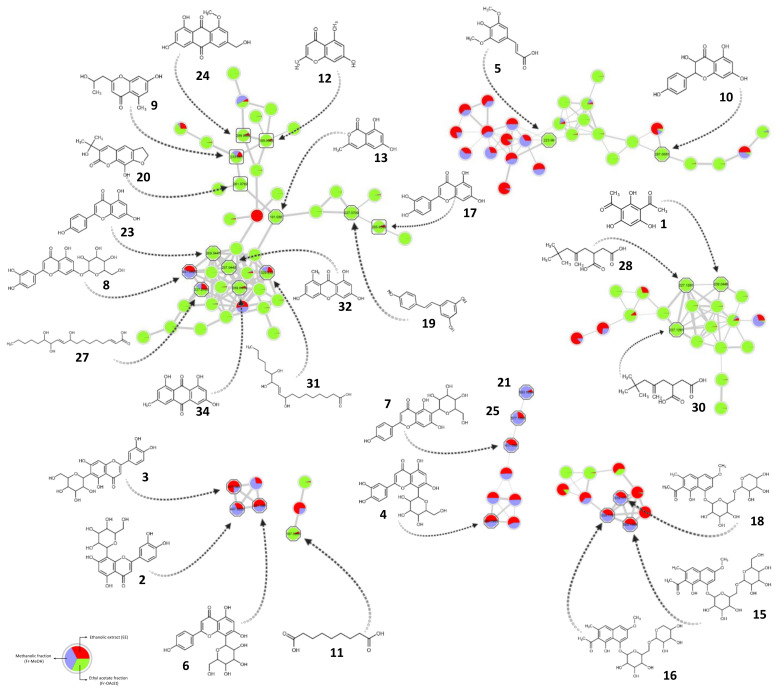
Selected clusters and putative identified compounds from the molecular network in negative ion mode for the ethanolic extract (EE), methanolic (Fr-MeOH) and ethyl acetate (Fr-OAcEt) fractions of *Chamaecrista diphylla* leaves. Numbers inside the nodes correspond to the accurate mass (*m*/*z*) for the [M–H]^–^ for each precursor. Node pie charts represent the proportion of each feature in EE (red), Fr-MeOH (blue) and Fr-OAcEt (green). Square shapes correspond to the isolated compounds. Octagonal shapes correspond to a match against an experimental or in silico MS^2^.

The excellent ORAC and total phenolic and total flavonoid contents obtained for the ethyl acetate enriched fraction justified an in-depth phytochemical investigation to identify the compounds responsible for the observed results. Dereplication of EE and fractions was performed by means of UHPLC-HRMS/MS in negative ionization mode This mode was preferred since it yielded a more sensitive detection of phenolic compounds. After data treatment using MZmine software [36], results were uploaded to the Global Natural Products Social Molecular Networking web platform GNPS [37]. The molecular network created contained 571 nodes with 22 hits from the GNPS libraries (Figure S2, Supplementary Information). Further identifications were obtained by dereplication against a large in silico spectra database of natural products [38], taxonomically informed reconsideration of the putative identities [39] and by manual comparison of the experimental fragmentation spectra against the data reported in the literature (in-house database, see Table S1 Supplementary Information) and other databases like MassBank [41] and MoNA (https://mona.fiehnlab.ucdavis.edu/). After this dereplication process, a total of 35 compounds were putatively identified as phenols (1, 19), flavones (2-4 6-8, 14, 21, 23, 25), phenolic acids (5), flavanonols (10), carboxylic acids (11, 27, 28, 30, 31, 35), isocoumarins (13), naphthalene derivatives (15, 16, 18), flavanones (22), anthraquinones (34) and xanthones (32) (see Table 2 and Figure 1). Structural chromophores were further confirmed using the UV data obtained from the UHPLC-PDA-HRMS runs. The positions of the compounds in all the chromatograms are shown in Figure S3 (Supplementary Materials).

All putatively identified compounds were present in the ethanolic extract (see Table 1). As expected after the chromatographic column enrichment procedure, certain compounds were concentrated in the methanolic or the ethyl acetate fractions. According to the putative identities, Fr-MeOH contained mostly flavones, flavanones and flavanonol derivatives. As expected, and in accordance with the chromatographic traces shown in Figure S1 (Supplementary Information), Fr-OAcEt contained mainly chromones, coumarins and anthraquinones derivatives, and less polar compounds. To further identify and corroborate the identity of as many compounds as possible, semi-preparative chromatographic separation allowed the isolation of eight compounds (9, 12, 17, 20, 26, 24, 29 and 33) from Fr-OAcEt. The compounds were analyzed in the same chromatographic and spectrometric conditions as the EE and fractions, corroborating their retention times, MS^2^ spectra and the putative structure initially proposed. No further structural analysis was performed on these compounds; thus, their identities remain putative. Compound 29 was left out of the initial molecular network analysis because it was lacking a high-quality fragmentation spectrum, and during the data processing it was discarded. Because the semi-preparative separation was based on the UV traces, the major peaks were collected, including 29, putatively identified as a rutaretin isomer, and consequently added to Table 1.

To corroborate the 35 putative identities, their fragmentation pattern was carefully examined and explained, following the fragmentation pathways reported in the literature according to their structural properties. For example, compounds 2 and 3 were putatively identified as orientin and isoorientin. These compounds were differentiated by the intensity of the fragment of *m*/*z* 357, at least 10% more intense for the orientin, and their chromatographic behavior. According to the literature, orientin elutes first in similar chromatographic conditions, solvents and chromatographic phases (see Figure S3, Supplementary Information) [42,43].

Similarly, compounds 6 and 7 were putatively identified as vitexin and isovitexin, based on mass spectra and the order of retention times, similar to those previously reported in the literature [43]. These molecules are analogous to 2 and 3, with one less hydroxyl in C-5. The fragmentation patterns are quite similar and share common fragmentation pathways. The fragment of *m*/*z* 429 for 2 and 3, and *m*/*z* 413 for compounds 6 and 7 correspond to dehydration [(M – H) – H_2_O]^–^, which is characteristic of the hexose unsaturation process [42,44,45,46,47,48]. Ions of *m*/*z* 369 (2,3) and *m*/*z* 353 (6,7) are likely produced through a ^0,4^X_1_ fragmentation of the remaining hexose moiety [(M – H) – H_2_O – C_2_H_4_O_2_]^−^. Further fragmentations take place in the hexoside unit, generating ions of *m*/*z* 357 (2,3) and *m*/*z* 341 (6,7) [(M – H) – H_2_O – C_3_H_4_O_2_]^−^ (^0,2^X_1_). A retro-Diels–Alder (RDA) ring cleavage [(M – H) – H_2_O – C_4_H_6_O_3_)]^−^ (^0,3^X_1_) leads to the fragments of *m*/*z* 327 (2,3) and *m*/*z* 311 (6,7). The loss of carbon monoxide was observed for all the compounds, [(M – H) – H_2_O – C_4_H_6_O_3_ – CO]^–^ (^0,1^X_1_), the ion of *m*/*z* 299 (2,3) and *m*/*z* 283 (6,7).

The molecular network (Figure 1) shows 21 clustering with compounds 7 (isovitexin) and 25 (isovitexin-*O*-pentoside). This is likely due to the neutral losses occurring in the glycoside and flavone units, as discussed above. However, the fragmentation pattern observed for 21 unequivocally suggests an orientin as the aglycone core, rather than isovitexin, as for 7 and 25. According to the difference observed between the parent ions and the aglycone fragments, and several other fragments, the glycosides correspond to a hexoside and a pentoside for 7 and 25, respectively.

Three possible rutaretin isomers [49] were detected at different retention times, with the same fragmentation patterns (20, 26, 29). Two consecutive losses of a methyl radical were observed, producing the ions of *m*/*z* 246 and *m*/*z* 231. These two methyls are part of the CH_3_(CO)CH_3_ group, confirmed after the loss of an acetone molecule and generation of *m*/*z* 217. The loss of CO_2_ from the B ring, characteristic of coumarins, was observed [50]. Additionally, loss of CH_2_O produced *m*/*z* 231, confirming the presence of the 2,3-dihydrofuran core. These isomers could correspond to variations of the hydroxylation pattern in positions C-4, C-6 and C-9. Further isolation processes and spectroscopic analyses are needed to corroborate this hypothesis. Detailed fragmentation pathway explanations for all the other compounds can be found in the Supplementary Information.

### 3.3. Antioxidant Activity Evaluation of the Isolated Compounds

**Figure 2:**
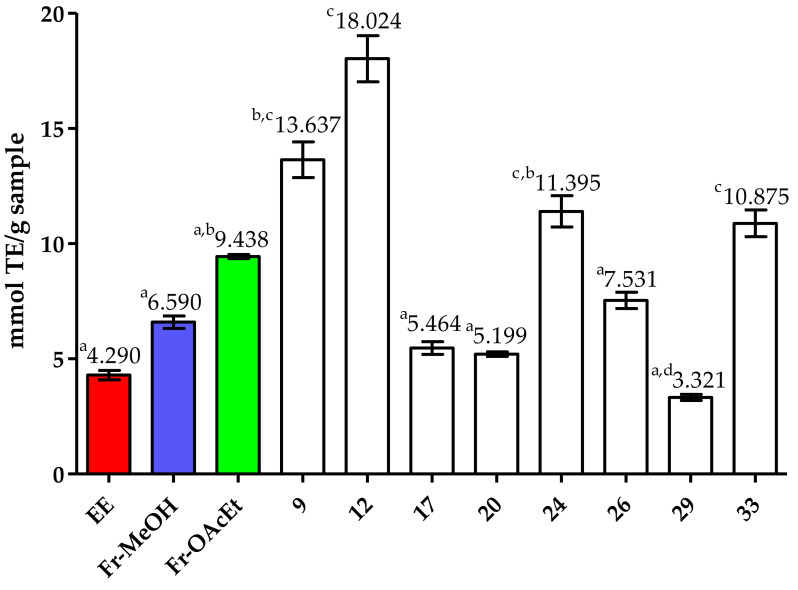
Oxygen radical absorbance capacity comparison for the ethanolic extract (EE), Fr-MeOH, Fr-OAcEt and the 8 isolated compounds from the leaves of *Chamaecrista diphylla*. The Kruskal–Wallis non-parametric test (equivalent to ANOVA) and Dunn’s post-test were applied [60]. The tests were based on the statistical requisites available in the literature [61] performed in the software R v. 4.03. Different letters refer to significant differences between the eleven samples (EE, Fr-MeOH, Fr-OAcEt, 9, 12, 17, 20, 24, 26, 29, and 33) at a level of *p* < 0.05.

The antioxidant capacity for the eight isolated compounds was evaluated through the ORAC assay (See Figure 2). As mentioned above, Fr-OAcEt had important activity (9.44 ± 0.09 mmol TE/g) relative to Fr-MeOH and the original ethanolic extract (EE). As expected, some of the compounds isolated from Fr-OAcEt had a considerable antioxidant capacity, higher than that of Fr-OAcEt (9, 12, 24 and 33). According to their putative identity, these results agree with the reports in the literature for their chemical classes [53,54,55]. The nominal ORAC indexes for 9 and 12 were the highest, and these are chromones-like structures, a chemical class reported already to have high antioxidant activity [56,57,58,59].

### 3.4. Bond Dissociation Enthalpy (BDE) Calculations for Selected Compounds

The bond dissociation enthalpy (BDE) of the O–H bond is strongly correlated with the antioxidant activity capacity, as some hydroxy-containing compounds usually donate a hydrogen atom to the free radicals, thus neutralizing the toxic effect [62,63]. It is possible to determine the BDE values as the difference in the heat of formation between the neutral molecule and its radical (the BDE corresponds to the O-H bond-breaking energy). The BDE values were calculated considering the solvation effects (water and methanol) and the radical position (regarding the structure) for the eight isolated compounds (See Table 3). The calculations showed that 12 and 9 require lower energy to break the O-H bond in positions 7-O^•^ than the analog compounds, sustaining the antioxidant result observed. Additionally, the solvation effect computed for the BDE showed a higher preference for bond-breaking in water than in methanol. For the rutaretin and the carviolin derivatives, a higher energy, more than 100 kcal/mol, is needed to dissociate the O-H bond, which correlates with their observed lower antioxidant capacity.

## 4. Discussion

Prospecting for plants that are rich in phenolic compounds is supported by studies highlighting the strong correlation between phenolic content and antioxidant capacity [64]. Furthermore, compounds exhibiting an antioxidant capacity tend to present other activities, such as anti-diabetic [65], anti-inflammatory [66], antibacterial [67,68,69] and antiproliferative activities [70]. Determining the antioxidant capacity by means of the ORAC assay is one of the best methods currently used [71,72]. This assay uses the radical peroxyl, a reactive oxygen species (ROS) present in the human body. Some phenolics have been shown to increase the total antioxidant capacity of plasma. There is a strong dependence on their dietary intake, bio-availability and metabolization [73].

In the current study, we found that the ethanolic extract of *Chamaecrista diphylla* presents significant antioxidant activity, as well as a high content of phenolics and flavonoids. A chromatographic enrichment procedure allowed the generation of two fractions, with greater antioxidant, flavonoid and phenolic content values, and differentiated chromatographic profiles. This simplified the complexity of the mixture, helped to locate the antioxidant activity and enhanced the putative identification rate. These results show that the total phenolic and flavonoid contents for Fr-OAcEt were approximately 2-fold greater compared to EE and Fr-MeOH. This was due to the removal of small hydrophilic molecules like carbohydrates and other polar compounds in the fractionation process. The important antioxidant potential of Fr-OAcEt is comparable to those reported for several plants of pharmacological importance, such as *Limonium densiflorum*, a plant rich in phenolics with antioxidant (4.48 ± 0.25 µmol TE/g sample), anti-inflammatory and anti-cancer activities in human cell lines [74]. *Prunus persica* (3.43 ± 0.09 mmol TE/g sample) shows strong anti-lipase and anti-dementia activities [75]. *Bursera microphylla* (1.63 ± 0.05 mmol TE/g sample) is reported to show important anti-inflammatory activity [76].

To gain further insights into the bioactive phytochemicals responsible for those antioxidant activities, Fr-OAcEt was subjected to isolation, yielding eight pure compounds (9, 12, 17, 20, 26, 24, 29 and 33). The nominal antioxidant capacities of these compounds highlighted 9, 12, 24 and 33 as those responsible for the high antioxidant results shown for Fr-OAcEt, and these probably contribute greatly to the EE overall activity. To identify these compounds and as many others as possible, liquid chromatography coupled with tandem mass spectrometry (UPLC-HRMS/MS) analysis, combined with molecular networking data processing, was applied to the EE and its derived fractions. Interestingly, 35 compounds were putatively identified, mostly flavonoids and derivatives. All of them are reported for the first time in the species and thirty-two (1–16, 18–22, 24–33 and 35) are reported for the first time in the genus.

In Fr-OAcEt, we observed a predominance of chromones, isocoumarins, coumarins, anthraquinones and carboxylic acids. Furthermore, four flavonoids were found—dihydrokaempferol (10), luteolin (17), naringenin (22) and apigenin (23). Their presence in this fraction can be explained by their lower polarity. The flavonoid content value for Fr-OAcEt is in accordance with the presence of compounds such as dihydrokaempferol (10), luteolin (17), naringenin (22) and apigenin (23), which are present in greater concentrations in relation to the flavonoids (2, 3, 4, 6, 7 and 8) in Fr-MeOH. The antioxidant capacity for Fr-MeOH could be directly related to the presence of flavonoids such as orientin (2), isoorientin (3), vitexin (6), isovitexin (7), luteolin-7-*O*-glucoside (8) and luteolin (17), all of which are reported as antioxidant compounds [77,78,79,80,81]. According to the proportion of each feature in the different fractions represented in the molecular network, only a few peaks were shared between them. The largest clusters in the molecular network were almost completely specific to Fr-OAcEt, and when the clusters included ions from both fractions, their fraction specificity was high. This means that a cluster could be formed for ions belonging to both fractions, but the presence of the respective ions is mainly in one or another fraction. The chemistry across the clusters is well sustained and homogenous.

The antioxidant activity of molecules is related to their capacity to donate hydrogen atoms or electrons through to the O–H bond dissociation [56,57,58,59]. BDE calculations can be used to correlate the antioxidant capacity observed with the structural features [82]. Thus, the lower the BDE value, the greater the antioxidant potential. The BDE results obtained for the isolated compounds agree with the experimental results for antioxidant capacity. Compounds 9 and 12 bear a chromenone-core structure, whereas 24 and 33 are anthraquinones. Both these types of structure have been already reported to have potent antioxidant capacities [56,57,58,59,83,84,85]. These four promising antioxidant compounds could present other interesting properties. They belong to chemical classes reported in the literature to have anti-inflammatory [86], antiproliferative [87] and antidiabetic activities [84], as well as some uses against breast cancer [88]. Therefore, most of the results obtained in the leaves of *C. diphylla* reveal it to be a potential natural source of antioxidants, although further studies are still needed to understand its pharmacological potential in vivo.

## 5. Conclusions

In this study, we used UHPLC-MS/MS data, combined with the molecular network approach, to putatively identify compounds from an extract and enriched fractions of *C. diphylla* leaves with potent antioxidant activity. The chemical annotation was considerably expanded with the use of FBMN-GNPS—a total of thirty-five compounds were characterized and attributed to various chemical classes, all of which were reported for the first time in the species and thirty-two for the first time in the genus. Eight compounds were isolated from Fr-OAcEt and compounds 9, 12, 24 and 33 showed excellent antioxidant capacities. These results suggest that *C. diphylla* is a natural source of antioxidant compounds, which is interesting for future biological studies.

## Figures and Tables

**Table 1:** Antioxidant activity (ORAC), total phenolic and total flavonoids contents for *Chamaecrista diphylla* leaves ethanolic extract (EE), Fr-MeOH and Fr-OAcEt.

Samples	AA (mmol TE/g)	TP (mg GAE/g)	TF (mg CE/g)
	ORAC Value		
Ethanolic extract (EE)	4.29 ± 0.20	131.08 ± 0.95	9.31 ± 0.26
Fr-MeOH	6.59 ± 0.27	157.38 ± 13.20	9.58 ± 0.12
Fr-OAcEt	9.44 ± 0.09	302.21 ± 3.10	21.9 ± 0.10

AA: antioxidant activity; TP: total phenols; TF: total flavonoids; TE: Trolox equivalent; GAE: gallic acid equivalent; CE: catechin equivalent.

**Table 2:** Putative identified chemical constituents of *Chamaecrista diphylla* extract and fractions.

Peak	RT (min)	MF	[M–H]^–^			Product Ions MS/MS	Putative Identity	Reference Spectrum ID	EE	Fr-MeOH	Fr-OAcEt
			Theoretical Accurate *m/z*	Experimental Accurate *m/z*	Error ppm						
1	4.10	C_10_H_10_O_5_	209.0450	209.0446	1.9	191, 165, 123	2,4-diacetylphloroglucinol	^b^ CCMSLIB00004693587	x	-	x
2	4.36	C_21_H_20_O_11_	447.0927	447.0925	0.4	429, 369, 357, 327, 299, 285	orientin	^c^ FIO00705	x	x	-
3	4.48	C_21_H_20_O_11_	447.0927	447.0930	0.7	429, 369, 357, 327, 299, 285	isoorientin	^c^ FIO00715	x	x	-
4	4.61	C_22_H_22_O_13_	^a^ 493.0982	^a^ 493.0985	0.6	447 [M – H]^–^, 369, 357, 327, 299, 285	2-(3,4-dihydroxyphenyl)-5,7-dihydroxy-8-[3,4,5-trihydroxy-6-(hydroxymethyl)oxan-2-yl]chromen-4-one	^b^CCMSLIB00000846053	x	x	-
5	4.80	C_11_H_12_O_5_	223.0606	223.0600	2.7	208, 193, 179, 164, 149	sinapic acid	^b^CCMSLIB00005738417	x	-	x
6	5.02	C_21_H_20_O_10_	431.0978	431.0980	0.5	413, 353, 341, 311, 283, 269	vitexin	^d^FIO00915	x	x	-
7	5.12	C_21_H_20_O_10_	431.0978	431.0980	0.5	413, 353, 341, 311, 283, 269	isovitexin	^d^FIO00915	x	x	-
8	5.40	C_21_H_20_O_11_	447.0927	447.0925	0.4	357, 339, 327, 311, 299, 285, 255, 151, 133	luteolin-7-*O*-glucoside	^c^VFNPL-QEHF013327	x	x	-
9	5.68	C_13_H_14_O_4_	233.0814	233.0810	1.7	215, 189, 174 161, 149	Aloesol	[49]	x	x	x
10	5.93	C_15_H_12_O_6_	287.0556	287.0551	1.7	269, 259, 243, 201, 151	dihydrokaempferol	^b^CCMSLIB00004684095	x	-	x
11	6.27	C_9_H_16_O_4_	187.0970	187.0965	2.7	181, 125	azelaic acid	^c^ KO000123	x	-	x
12	6.36	C_11_H_10_O_3_	189.0552	189.0545	3.7	174, 161, 149	7-hydroxy-2,5-dimethyl-4H-chromen-4-one	[51]	x	x	x
13	6.40	C_10_H_8_O_4_	191.0344	191.0360	8.4	176, 149	1H-2-benzopyran-1-one, 6,8-dihydroxy-3-methyl	^c^ VF-NPL-QEHF001189	x	-	x
14	6.61	C_22_H_22_O_13_	461.1084	461.1072	2.6	446, 299	5-[4-[(2S,3R,4S,5S,6R)-3,4,5-trihydroxy-6-(hydroxymethyl)oxan-2-yl]oxyphenyl]chromen-4-one	^b^ CCMSLIB00004717546	x	x	-
15	7.16	C_26_H_34_O_14_	569.1870	569.1868	0.4	245, 230, 215	torachrysone-8-hexosyl-hexoside	^e^ HOV19-D	x	x	-
16	7.57	C_25_H_32_O_13_	539.1765	539.1760	0.9	245, 230, 215	torachrysone-8-hexosyl-pentoside isomer	^e^ HOV18-C	x	x	-
17	7.72	C_15_H_10_O_6_	285.0399	285.0396	1.1	241, 217, 133	luteolin	^b^ CCMSLIB00004718183	x	x	x
18	7.80	C_25_H_32_O_13_	539.1765	539.1770	0.9	245, 230, 215	torachrysone-8-hexosyl-pentoside isomer	^e^ HOV18-C	x	x	-
19	8.10	C_14_H_12_O_3_	227.0708	227.0709	0.4	185, 157	resveratrol	^d^ BML00673	x	-	x
20	8.12	C_14_H_14_O_5_	261.0763	261.0762	0.4	246, 231, 217, 203	rutaretin isomer	[49]	x	-	x
21	8.46	C_30_H_26_O_13_	593.1295	593.1294	0.2	575, 447, 429, 369, 357, 327	orientin-*O*-hexoside	[43]	x	x	-
22	8.74	C_15_H_12_O_5_	271.0606	271.0601	1.8	253, 227, 199, 177, 151	naringenin	^b^ CCMSLIB00004719909	x	-	x
23	8.86	C_15_H_10_O_5_	269.0450	269.0447	1.1	225, 151	apigenin	^b^ CCMSLIB00005787970	x	-	x
24	9.22	C_16_H_12_O_6_	299.0556	299.0554	0.7	284, 256, 227, 199	carviolin isomer	^b^ CCMSLIB00004697562	x	x	x
25	9.25	C_30_H_26_O_12_	577.1346	577.1355	1.6	431, 413, 353, 341, 311, 283, 269	isovitexin-*O*-pentoside	[43]	x	x	-
26	9.90	C_14_H_14_O_5_	261.0763	261.0758	1.9	246, 231, 217, 203	rutaretin isomer	[49]	x	-	x
27	9.97	C_18_H_32_O_5_	327.2171	327.2165	1.8	291, 229, 211, 183, 171	9,12,13-trihydroxy-10(*E*),15(*Z*)- octadecadienoic acid	[52]	x	x	x
28	10.2	C_12_H_20_O_4_	227.1283	227.1281	0.9	209, 183, 165	butanedioic acid, 2-(4,4-dimethyl-2-methylenepentyl) isomer	^b^ CCMSLIB00003138678	x	-	x
29	10.3	C_14_H_14_O_5_	261.0763	261.0754	3.4	246, 231, 217, 203	rutaretin isomer	[49]	x	-	x
30	10.4	C_12_H_20_O_4_	227.1283	227.1281	0.9	209, 183, 165	butanedioic acid, 2-(4,4-dimethyl-2-methylenepentyl) isomer	^b^ CCMSLIB00003138678	x	-	x
31	10.8	C_18_H_34_O_5_	329.2328	329.2320	2.4	311, 293, 229, 211, 183, 171	9,12,13-trihydroxyoctadec-10-enoic acid	^e^ JTY13-R[52]	x	x	-
32	10.9	C_14_H_10_O_5_	257.0450	257.0442	3.1	213, 171, 159, 137	norlichexanthone	^c^ VFNPL-QEHF017949	x	-	x
33	11.2	C_16_H_12_O_6_	299.0556	299.0550	2.0	284, 256, 227, 199	carviolin isomer	^b^ CCMSLIB00004697562	x	x	-
34	14.3	C_15_H_10_O_5_	269.0450	269.0444	2.2	241, 225	emodin	^b^ CCMSLIB00004702275	x	-	x
35	16.6	C_18_H_30_O_3_	293.2117	293.2115	0.7	275, 256, 224, 195	hydroxyoctadecatrienoic acid	^d^ IA000196 13-HOTrE	x	-	x

Note: RT: Retention time; MF: Molecular formula; ^a^ [M + HCOOH–H]^–^; spectrum ID in ^b^ GNPS (https://gnps.ucsd.edu/); spectrum ID in ^c^ MoNA (https://mona.fiehnlab.ucdavis.edu/); spectrum ID in ^d^ MassBank (http://massbank.jp/); ^e^ compound ID in the Dictionary of Natural Products (CRC number, this is a match against the in silico database); EE: ethanolic extract; Fr-MeOH: methanolic fraction; Fr-OAcEt: ethyl acetate fraction.

**Table 3:** Bond dissociation enthalpies (BDEs) corresponding to the formation of radical species for the isolated compounds in this study.

Radical Species	BDE/kcal mol^−1^
*Chromones*	
aloesol (9)	
7-O^•^	89.3
	90.3 ^a^
	90.0 ^b^
12-O^•^	104.3
	105.1 ^a^
	105.1 ^b^
7-hydroxy-2,5-dimethyl-4H-chromen-4-one (12)	
7-O^•^	83.5
	89.3 ^a^
	120.8 ^b^
*Flavone*	
luteolin (17)	
5-O^•^	103.8
6-O^•^	90.5
4′-O^•^	85.9
5′-O^•^	78.0
*Coumarin and anthraquinone isomers*	
rutaretin (20, 26, 29)	
(CH_3_)-O^•^	107.3
8-O^•^	121.5
carviolin (24, 33)	
1-O^•^	108.1
3-O^•^	90.7
6-CH_2_-O^•^	100.7

Note: ^a^: BDE calculated in water; ^b^: BDE calculated in methanol.

## Data Availability

All support data used in this study are available from the authors.

## Supplementary Materials

The following are available online at https://www.mdpi.com/article/10.3390/pharmaceutics13050681/s1, Figure S1. Total ion chromatograms, in negative ionization mode, for ethanolic extract (EE), ethyl acetate fraction (Fr-OAcEt) and methanolic fraction (Fr-MeOH). Figure S2. Combined Molecular network for the ethanolic extract (red), methanolic fraction (blue) and ethyl acetate fraction (green) of *Chamaecrista diphylla* leaves. Figure S3. UHPLC-MS total ion chromatograms (region 3.5 to 17 min) in negative ion mode for *Stryphnodendron pulcherrimum* leaf extract, showing the 35 dereplicated compounds. (A) Ethanolic extract (EE). (B) Ethyl acetate fraction (Fr-OAcEt). (C) Methanolic fraction (Fr-MeOH). Table S1. In-house database of compounds reported in the Genus *Chamaecrista*. References [89,90,91,92,93,94,95,96,97,98,99,100,101,102,103,104,105,106,107,108,109,110,111,112,113,114,115,116,117,118,119,120,121,122,123,124,125,126,127,128,129,130] are cited in the supplementary materials.
