# Supplementary material for: Constituents of Chamaecrista diphylla (L.) Greene Leaves with Potent Antioxidant Capacity: A Feature-Based Molecular Network Dereplication Approach
Source: Pharmaceutics. 2021 May 10;13(5):681. doi: 10.3390/pharmaceutics13050681 (PMC8150882; doi:10.3390/pharmaceutics13050681)
Supplement: Supplementary file 1 [file pharmaceutics-13-00681-s001.zip › pharmaceutics-1170778 - supplementary.pdf]

pharmaceutics

Supplementary Materials: Constituents of *Chamaecrista diphylla* (L.) Greene Leaves with Potent Antioxidant Activity: A Feature-Based Molecular Network Dereplication Approach

Paulo Gomes, Luis Quirós-Guerrero, Abraão Muribeca, José Reis, Sônia Pamplona, Anderson H. Lima, Mariele Trindade, Consuelo Silva, Jesus Souza, Jean Bouti, Jean-Luc Wolfender and Milton Silva

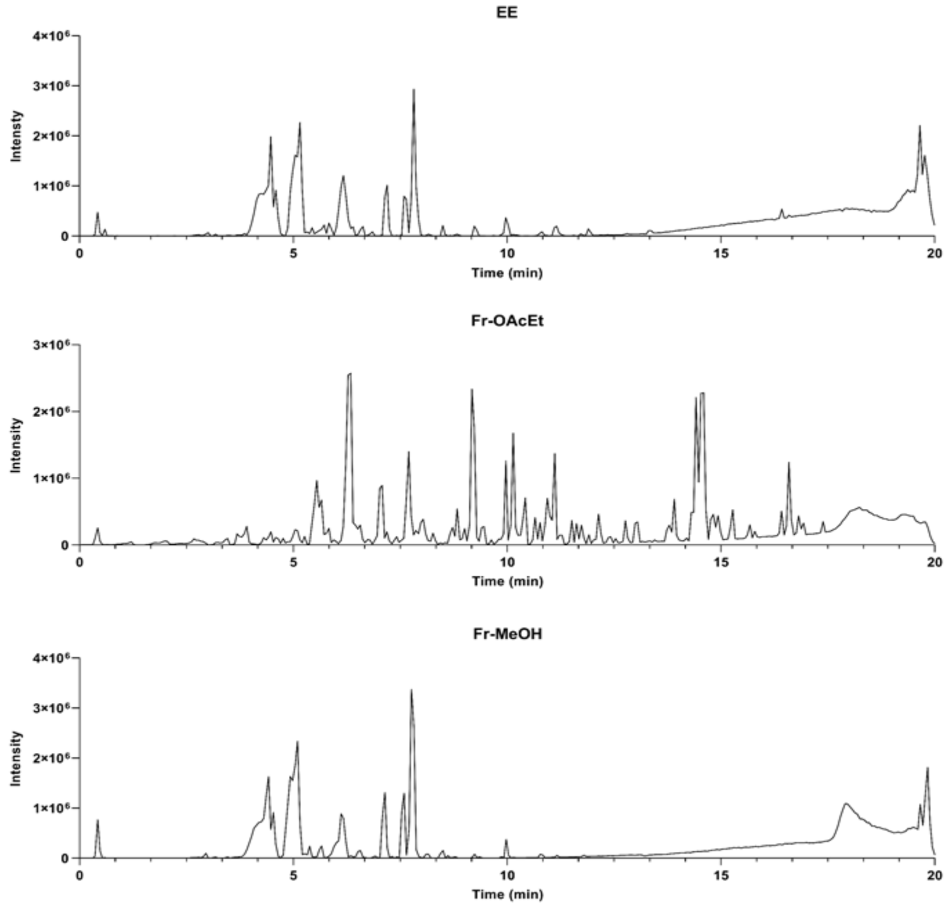

Figure S1. Total ion chromatograms in negative ionization mode, for ethanolic extract (EE), ethyl acetate fraction (Fr-OAcEt) and methanolic fraction (Fr-MeOH).

Publisher's Note: MDPI stays neutral with regard to jurisdictional claims in published maps and institutional affiliations.

Copyright: © 2020 by the authors. Submitted for possible open access publication under the terms and conditions of the Creative Commons Attribution (CC BY) license (http://creativecommons.org/licenses/by/4.0/).

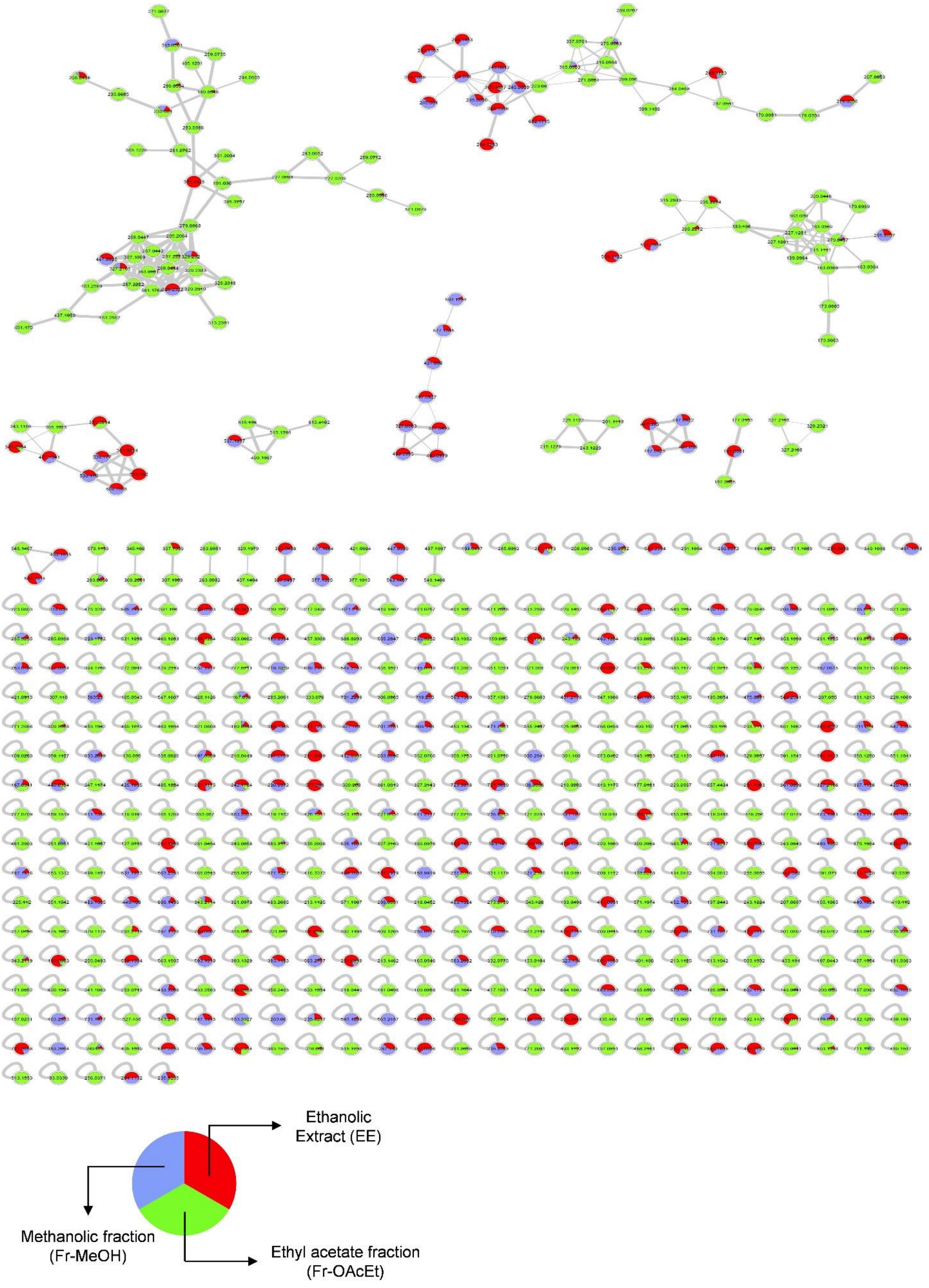

Figure S2. Combined molecular network for the ethanolic extract (red), methanolic fraction (blue) and ethyl acetate fraction (green) of *Chamaecrista diphylla* leaves. Pie charts inside the nodes represent the proportion of each feature in the extract and fractions. Numbers inside each node correspond to the precursor ion of the feature.

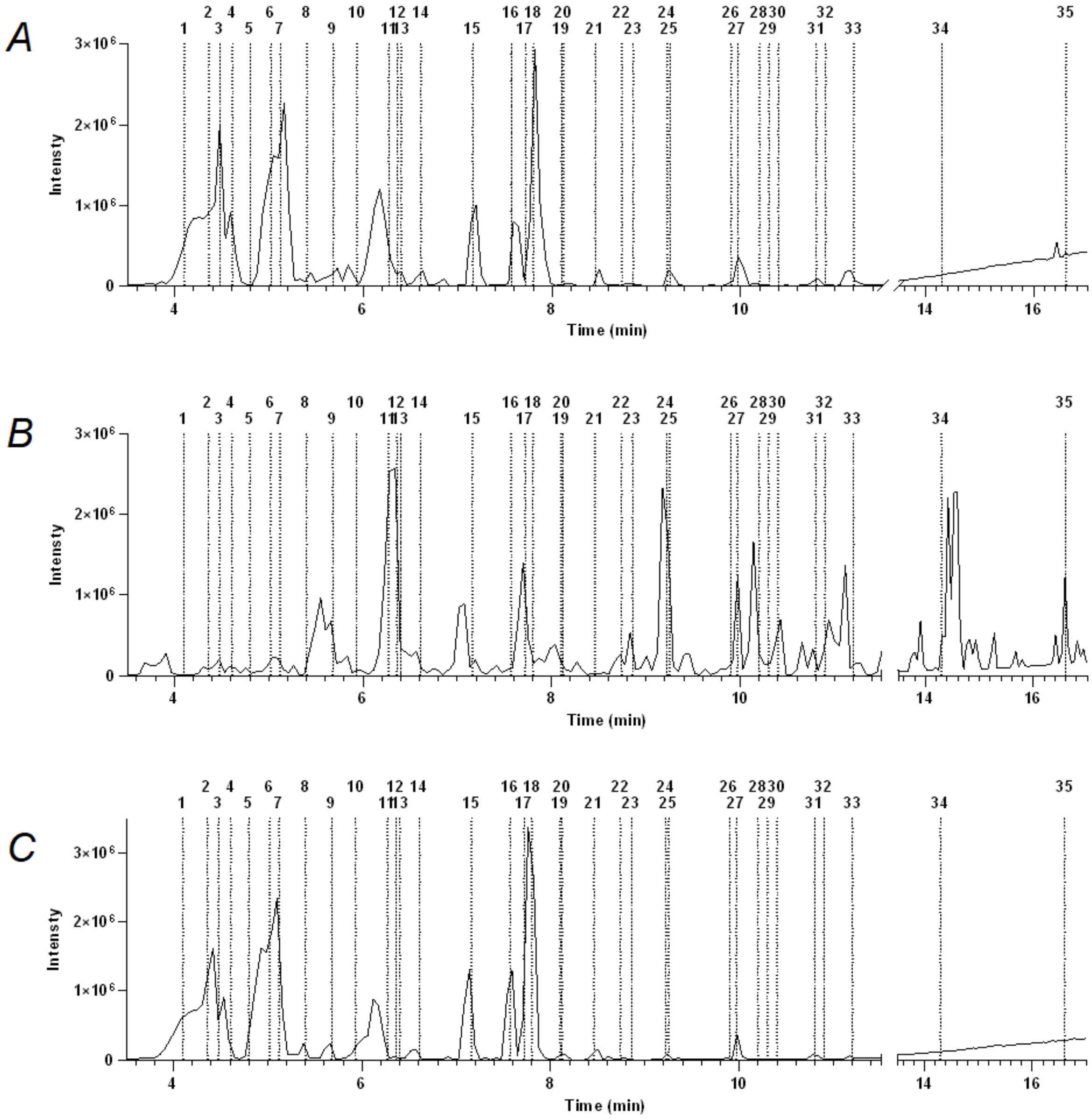

Figure 3. UHPLC–MS total ion chromatograms (region 3.5–17 min) in the negative ion mode for the *Stryphnodendron pulcherrimum* leaf extract showing the 35 dereplicated compounds: (**A**) ethanolic extract (EE); **(B)** ethyl acetate fraction (Fr-OAcEt); and (**C**) methanolic fraction (Fr-MeOH). The region between 11.5 and 13.5 is not shown here for better visualization (there were no compounds putatively identified here). The total chromatographic traces can be seen in Figure S1.

Table S1. In-house database of compounds reported in the genus *Chamaecrista*.

ID	Compound	MF	(M – H)$^-$ Calculated	Species	Reference
1	(Epi)fis-(epi)fis-(epi)cat-(epi)fis	$C_{60}H_{50}O_{21}$	1105.2766	*Chamaecrista nictitans*	[30]
2	(Epi)fis-(epi)cat-(epi)afz-(epi)gui	$C_{60}H_{50}O_{20}$	1089.2817	*Chamaecrista nictitans*	[30]
3	(Epi)gui-(epi)afz-(epi)fis-(epi)cat	$C_{60}H_{50}O_{20}$	1089.2817	*Chamaecrista nictitans*	[30]
4	(Epi)fis-(epi)fis-(epi)fis-(epi)fis	$C_{60}H_{50}O_{20}$	1089.2817	*Chamaecrista nictitans*	[30]
5	(Epi)gui-(epi)fis-(epi)fis-(epi)fis	$C_{60}H_{50}O_{19}$	1073.2868	*Chamaecrista nictitans*	[30]

6	(Epi)afz-(epi)afz-(epi)fis-(epi)gui	$C_{60}H_{50}O_{19}$	1073.2868	*Chamaecrista nictitans*	[30]
7	(Epi)gui-(epi)cat-(epi)gui-(epi)gui	$C_{60}H_{50}O_{18}$	1057.2919	*Chamaecrista nictitans*	[30]
8	(Epi)cat-(epi)fis-(epi)fis	$C_{45}H_{38}O_{16}$	833.2082	*Chamaecrista nictitans*	[30]
9	(Epi)fis-(epi)cat-(epi)fis	$C_{45}H_{38}O_{16}$	833.2082	*Chamaecrista nictitans*	[30]
10	(Epi)afz-(epi)cat-(epi)afz	$C_{45}H_{38}O_{16}$	833.2082	*Chamaecrista nictitans*	[30]
11	(Epi)gui-(epi)cat-(epi)cat	$C_{45}H_{38}O_{16}$	833.2082	*Chamaecrista nictitans*	[30]
12	(Epi)fis-(epi)cat-(epi)gui	$C_{45}H_{38}O_{15}$	817.2132	*Chamaecrista nictitans*	[30]
13	(Epi)gui-(epi)cat-(epi)fis	$C_{45}H_{38}O_{15}$	817.2132	*Chamaecrista nictitans*	[30]
14	(Epi)afz-(epi)fis-(epi)fis	$C_{45}H_{38}O_{15}$	817.2132	*Chamaecrista nictitans*	[30]
15	(Epi)fis-(epi)fis-A-(epi)afz	$C_{45}H_{36}O_{15}$	815.1976	*Chamaecrista nictitans*	[30,31]
16	(Epi)fis-A-(epi)fis-(epi)afz	$C_{45}H_{36}O_{15}$	815.1976	*Chamaecrista nictitans*	[30]
17	(Epi)gui-(epi)fis-(epi)fis	$C_{45}H_{38}O_{14}$	801.2183	*Chamaecrista nictitans*	[30]
18	(Epi)fis-(epi)fis-(epi)gui	$C_{45}H_{38}O_{14}$	801.2183	*Chamaecrista nictitans*	[30,31]
19	(Epi)fis-(epi)afz-(epi)gui	$C_{45}H_{38}O_{14}$	801.2183	*Chamaecrista nictitans*	[30]
20	(Epi)gui-(epi)cat-(epi)gui	$C_{45}H_{38}O_{14}$	801.2183	*Chamaecrista nictitans*	[30]
21	Kaempferol-*O*-hexoside-*O*-rhamnosylhexoside	$C_{33}H_{40}O_{20}$	755.2035	*Chamaecrista nictitans*	[30]
22	Quercetin-*O*-rhamnoside-*O*-rhamnosylpentoside	$C_{32}H_{38}O_{19}$	725.1929	*Chamaecrista nictitans*	[30]
23	Rhamnetin-*O*-pentoside-hexoside-*O*-acetate	$C_{29}H_{32}O_{17}$	651.1561	*Chamaecrista nictitans*	[30]
24	Kaempferol-*O*-rhamnosylhexoside-*O*-acetate	$C_{29}H_{32}O_{16}$	635.1612	*Chamaecrista nictitans*	[30]
25	Rhamnetin-*O*-hexosylrhamnoside	$C_{28}H_{32}O_{16}$	623.1612	*Chamaecrista nictitans*	[30]
26	Kaempferol-*O*-(acetyl)hexosidepentoside	$C_{28}H_{30}O_{16}$	621.1456	*Chamaecrista nictitans*	[30]
27	Quercetin-*O*-rhamnosyl-(1→6)-hexoside	$C_{27}H_{30}O_{16}$	609.1456	*Chamaecrista nictitans*	[30]
28	Luteolin-6-*C*-hexosyl-(1→2)-rhamnoside	$C_{27}H_{30}O_{15}$	593.1506	*Chamaecrista nictitans*	[30]
29	Luteolin-6-*C*-hexosyl-(1→2)-rhamnoside isomer	$C_{27}H_{30}O_{15}$	593.1506	*Chamaecrista nictitans*	[30]
30	Kaempferol-6-*C*-hexosyl-(1→2)-rhamnoside	$C_{27}H_{30}O_{15}$	593.1506	*Chamaecrista nictitans*	[30]
31	Kaempferol-6-*C*-hexosyl-(1→2)-rhamnoside isomer	$C_{27}H_{30}O_{15}$	593.1506	*Chamaecrista nictitans*	[30]
32	Apigenin-6-*C*-hexosyl-(1→2)-rhamnoside	$C_{27}H_{30}O_{14}$	577.1557	*Chamaecrista nictitans*	[30]
33	(Epi)cat-(epi)cat	$C_{30}H_{26}O_{12}$	577.1346	*Chamaecrista nictitans*	[30]
34	Cassiaoccidentalin B	$C_{27}H_{28}O_{14}$	575.1401	*Chamaecrista nictitans*	[30,31]
35	Cassiaoccidentalin A	$C_{27}H_{28}O_{13}$	559.1452	*Chamaecrista nictitans*	[30]
36	Cassiaoccidentalin A isomer (1)	$C_{27}H_{28}O_{13}$	559.1452	*Chamaecrista nictitans*	[30]
37	Cassiaoccidentalin A isomer (2)	$C_{27}H_{28}O_{13}$	559.1452	*Chamaecrista nictitans*	[30]
38	(Epi)gui-(epi)cat	$C_{30}H_{26}O_{10}$	545.1448	*Chamaecrista nictitans*	[30]

39	Rhamnetin-*O*-(acetyl)hexoside	$C_{24}H_{24}O_{13}$	519.1139	*Chamaecrista nictitans*	[30]
40	Quercetin-*O*-hexoside-*O*-acetate	$C_{23}H_{22}O_{13}$	505.0982	*Chamaecrista nictitans*	[30]
41	Quercetin-*O*-hexoside	$C_{21}H_{20}O_{12}$	463.0877	*Chamaecrista nictitans*	[30]
42	Luteolin-*C*-hexoside	$C_{21}H_{20}O_{11}$	447.0929	*Chamaecrista nictitans*	[30]
43	Apigenin-*C*-hexoside	$C_{21}H_{20}O_{10}$	431.0978	*Chamaecrista nictitans*	[30]
44	Fisetin	$C_{15}H_{10}O_{6}$	285.0399	*Chamaecrista nictitans*	[30,31]
45	Luteolin	$C_{15}H_{10}O_{6}$	285.0399	*Chamaecrista nictitans*	[31]
46	Luteolin hexoside	$C_{21}H_{20}O_{11}$	447.0927	*Chamaecrista nictitans*	[31]
47	Luteolin dihexoside	$C_{27}H_{30}O_{16}$	609.1456	*Chamaecrista nictitans*	[31]
48	Ellagic acid	$C_{14}H_{6}O_{8}$	300.9984	*Chamaecrista nictitans*	[31]
49	Ellagic acid pentoside	$C_{19}H_{14}O_{12}$	433.0407	*Chamaecrista nictitans*	[31]
50	Quercetin hexoside	$C_{21}H_{20}O_{12}$	463.0877	*Chamaecrista nictitans*	[31]
51	Quercetin pentoside	$C_{20}H_{18}O_{11}$	433.0771	*Chamaecrista nictitans*	[31]
52	Apigenin	$C_{15}H_{10}O_{5}$	269.0450	*Chamaecrista nictitans*	[31]
53	Apigenin hexoside	$C_{21}H_{20}O_{10}$	431.0978	*Chamaecrista nictitans*	[31]
54	(Epi)gui-(epi)afz	$C_{30}H_{26}O_{9}$	529.1499	*Chamaecrista nictitans*	[31]
55	(Epi)fis-(epi)afz	$C_{30}H_{26}O_{10}$	545.1448	*Chamaecrista nictitans*	[31]
56	(Epi)fis-A-(epi)cat	$C_{30}H_{24}O_{11}$	559.1240	*Chamaecrista nictitans*	[31]
57	(Epi)fis-(epi)cat	$C_{30}H_{26}O_{11}$	561.1397	*Chamaecrista nictitans*	[31]
58	(Epi)fis-(epi)fis-(epi)afz	$C_{45}H_{38}O_{15}$	817.2132	*Chamaecrista nictitans*	[31]
59	(Epi)fis-(epi)cat-A-(epi)fis	$C_{45}H_{36}O_{16}$	831.1925	*Chamaecrista nictitans*	[31]
60	(Epi)cat-(epi)fis-A-(epi)afz	$C_{45}H_{36}O_{16}$	831.1925	*Chamaecrista nictitans*	[31]
61	(Epi)afz-(epi)afz-A-(epi)cat	$C_{45}H_{36}O_{16}$	831.1925	*Chamaecrista nictitans*	[31]
62	(Epi)fis-(epi)fis-(epi)cat	$C_{45}H_{38}O_{16}$	833.2082	*Chamaecrista nictitans*	[31]
63	Diisooctyl ester 1, 2-benzenedicarboxylic acid	$C_{24}H_{38}O_{4}$	389.2692	*Chamaecrista nigricans*	[32]
64	Methyl ester, (*Z*, *Z*, *Z*)-9, 12, 15-octadecatrienoic acid	$C_{19}H_{32}O_{2}$	291.2324	*Chamaecrista nigricans*	[32]
65	Nitric acid nonyl ester	$C_{9}H_{19}NO_{3}$	188.1287	*Chamaecrista nigricans*	[32]
66	4-C-Methyl-myo-inositol	$C_{7}H_{14}O_{6}$	193.0712	*Chamaecrista nigricans*	[32]
67	n-Hexadecanoic acid	$C_{16}H_{32}O_{2}$	255.2324	*Chamaecrista nigricans*	[32]
	2-Methyl-butanoic acid	$C_{5}H_{10}O_{2}$	101.0603	*Chamaecrista nigricans*	[32]
68	Octadecanoic acid	$C_{18}H_{36}O_{2}$	283.2637	*Chamaecrista nigricans*	[32]
69	Cholesterol	$C_{27}H_{46}O$	385.3470	*Chamaecrista absus*	[33]
70	Campesterol	$C_{28}H_{48}O$	399.3627	*Chamaecrista absus*	[33]
71	Stigmasterol	$C_{29}H_{48}O$	411.3627	*Chamaecrista absus*	[33]
72	β Sitosterol	$C_{29}H_{50}O$	413.3783	*Chamaecrista absus*	[33]

73	Δ-5-24, Stigmastadienol	$C_{29}H_{48}O$	411.3627	*Chamaecrista absus*	[33]
74	Δ-5-Avenasterol	$C_{29}H_{48}O$	411.3627	*Chamaecrista absus*	[33]
75	β Amyrin	$C_{30}H_{50}O$	425.3783	*Chamaecrista absus*	[33]
76	Cycloartenol	$C_{30}H_{50}O$	425.3783	*Chamaecrista absus*	[33]
77	24-Methylene-cycloartenol	$C_{31}H_{52}O$	439.3940	*Chamaecrista absus*	[33]
78	Chamaetexanin A	$C_{20}H_{28}O_3$	315.1960	*Chamaecrista flexuosa* *Chamaecrista greggii*	[34,89]
79	1,6,6,9a-tetramethyl-11-oxo-3b,4,5,5a,6,7,8,9,9a,9b,10,11-dodecahydrophenanthro[1,2-*c*]furan-4-yl acetate	$C_{22}H_{30}O_4$	357.2066	*Chamaecrista flexuosa*	[89]
80	Chamaetexanin B	$C_{20}H_{28}O_4$	331.1909	*Chamaecrista flexuosa* *Chamaecrista greggii*	[34,89]
81	(4-acetoxy-6,6,9a-trimethyl-11-oxo-3b,4,5,5a,6,7,8,9,9a,9b,10,11-dodecahydrophenanthro[1,2-*c*]furan-1-yl) methyl acetate	$C_{24}H_{32}O_6$	415.2121	*Chamaecrista flexuosa*	[89]
82	Chamaetexanin C	$C_{20}H_{30}O_3$	317.2117	*Chamaecrista flexuosa* *Chamaecrista greggii*	[34,89]
83	(4-acetoxy-4b,8,8-trimethyl-3-oxo-2-vinyl-3,4,4b,5,6,7,8,8a,9,10,10a-dodecahydrophenanthren-1-yl) methyl acetate	$C_{24}H_{34}O_5$	401.2328	*Chamaecrista flexuosa*	[89]
84	Chamaetexanin D	$C_{21}H_{30}O_4$	345.2066	*Chamaecrista flexuosa* *Chamaecrista greggii*	[34,89]
85	2-ethynyl-1-(methoxycarbonyl)-4b,8,8-trimethyl-3,4,4a,4b,5,6,7,8,8a,9,10,10a-dodecahydrophenanthrene-3,10-diyl diacetate	$C_{25}H_{34}O_6$	429.2277	*Chamaecrista flexuosa*	[89]
86	Chamaetexanin E	$C_{18}H_{24}O_3$	287.1647	*Chamaecrista flexuosa* *Chamaecrista greggii*	[34,89]
87	Chamaegreggane	$C_{21}H_{30}O_5$	361.2015	*Chamaecrista greggii*	[34]
88	Chamaetexane C acetonide	$C_{23}H_{34}O_3$	357.2430	*Chamaecrista greggii*	[34]
89	5-Hydroxy-1,4,6,7-tetramethoxy-2-methylanthraquinone	$C_{19}H_{18}O_7$	357.0974	*Chamaecrista greggii*	[34]
90	1,3,5-Trihydroxy-2,8-dimethoxy-6-methylanthracene-9,10-dione	$C_{17}H_{14}O_7$	329.0661	*Chamaecrista greggii*	[34]
91	1,2-Dihydroxy-3,5,8-trimethoxy-6-methylanthracene-9,10-dione	$C_{18}H_{16}O_7$	343.0818	*Chamaecrista greggii*	[34]
92	4,5-Dihydroxy-1,6,7-trimethoxy-2-methylanthraquinone	$C_{18}H_{16}O_7$	343.0818	*Chamaecrista greggii*	[34]
93	7-Hydroxy-4-10-dimethoxy-8-methylanthra[1,2-*d*][1,3]dioxole-6,11-dione	$C_{18}H_{14}O_7$	341.0661	*Chamaecrista greggii*	[34]
94	4-Hydroxy-1,5,6,7-tetramethoxy-2-methylanthraquinone	$C_{19}H_{18}O_7$	357.0974	*Chamaecrista greggii*	[34]
95	4,7-Dihydroxy-1,5,6-trimethoxy-2-methylanthraquinone	$C_{18}H_{16}O_7$	343.0818	*Chamaecrista greggii*	[34]
96	5,6-Dimethoxy-1,4,7-trihydroxy-2-methylanthraquinone	$C_{17}H_{14}O_7$	329.0661	*Chamaecrista greggii*	[34]
97	1,5-Dihydroxy-4,6,7-trimethoxy-2-methylanthraquinone	$C_{18}H_{16}O_7$	343.0818	*Chamaecrista greggii*	[34]
98	1,4,5,6,7-Pentamethoxy-2-methylanthraquinone	$C_{20}H_{20}O_7$	371.1131	*Chamaecrista greggii*	[34]

99	4,6-Dihydroxy-1,5,7-trimethoxy-2-methylanthraquinone	$C_{18}H_{16}O_7$	343.0818	*Chamaecrista greggii*	[34]
100	5,8-Dihydroxy-1,2,3-trimethoxy-6-methylanthracene-9,10-dione	$C_{18}H_{16}O_7$	343.0818	*Chamaecrista greggii*	[34]
101	1,7-Dimethoxy-4-hydroxy-5,6-methylenedioxy-2-methylanthraquinone	$C_{18}H_{14}O_7$	341.0661	*Chamaecrista greggii*	[34]
102	1,3-Dihydroxy-6-(hydroxymethyl)-2,5,8-trimethoxyanthracene-9,10-dione	$C_{18}H_{16}O_8$	359.0767	*Chamaecrista greggii*	[34]
103	4-Hydroxy-2-hydroxymethyl-1,5,6,7-tetramethoxyanthraquinone	$C_{19}H_{18}O_8$	373.0923	*Chamaecrista greggii*	[34]
104	4,6-Dihydroxy-2-hydroxymethyl-1,5,7-trimethoxyanthraquinone	$C_{18}H_{16}O_8$	359.0767	*Chamaecrista greggii*	[34]
105	4,7-Dihydroxy-2-hydroxymethyl-1,5,6-trimethoxyanthraquinone	$C_{18}H_{16}O_8$	359.0767	*Chamaecrista greggii*	[34]
106	1,2-Dihydroxy-3,8-dimethoxy-6-methylanthracene-9,10-dione	$C_{17}H_{14}O_6$	313.0712	*Chamaecrista greggii*	[34]
107	Physcion	$C_{16}H_{12}O_5$	284.0685	*Chamaecrista greggii*	[34]
108	Chrysophanol	$C_{15}H_{10}O_4$	253.0501	*Chamaecrista greggii*	[34]
109	4-Hydroxy-5,6,7-trimethoxy-2-methylanthraquinone	$C_{18}H_{16}O_6$	327.0869	*Chamaecrista greggii*	[34]
110	4,6-Dihydroxy-5,7-dimethoxy-2-methylanthraquinone	$C_{17}H_{14}O_6$	313.0717	*Chamaecrista greggii*	[34]
111	Betulinic acid	$C_{30}H_{48}O_3$	455.3525	*Chamaecrista greggii*	[34]
112	3-*O*-Betulinic acid p-coumarate	$C_{39}H_{54}O_5$	601.3893	*Chamaecrista greggii*	[34]
113	Pyracrenic acid	$C_{39}H_{54}O_6$	617.3842	*Chamaecrista greggii*	[34]
114	Lactone	$C_{17}H_{16}O_6$	315.0869	*Chamaecrista greggii*	[34]
115	4-(4-acetyl-3-methoxyphenyl)-2-(4-acetyphenyl)-5-oxotetrahydrofuran-3-yl acetate	$C_{23}H_{22}O_7$	409.1287	*Chamaecrista greggii*	[34]
116	Piceatannol	$C_{14}H_{12}O_4$	244.0736	*Chamaecrista greggii*	[34]
117	Resveratrol	$C_{14}H_{12}O_3$	227.0708	*Chamaecrista greggii*	[34]

1. Structural Identification of Compounds Present in the Extracts of C. diphylla

1.1. Phenol, Diphenol and Phenolic Acid Derivative

Literature reported other phloroglucinol derivatives in plants [90]. In addition, biosynthesis with acetyl-CoA and three molecules of malonyl-CoA shows that it is possible to form structures of floracetophenone [91]. This is evidenced with the phloracetophenone 4′-glucoside which has one less acetyl group and comprises one sugar linked [92]. Compound 1, $[M-H]^-$ at *m*/*z* 209.0446 was characterized as 2,4-diacetylphloroglucinol, with a spectral match against the GNPS libraries. Compound 5 at *m*/*z* 223.0600 was identified as sinapic acid. The consecutive loss of two methyl groups produced fragments at *m*/*z* 208 $[(M-H)-CH_3]^-$ and *m*/*z* 193 $[(M-H)-2CH_3]^-$. Additional decarboxylations from these two ions generated the ions at *m*/*z* 164 and *m*/*z* 149, respectively [93]. Compound 19, putatively identified as resveratrol, was detected at *m*/*z* 227.0709. The fragmentation pattern confirmed against the GNPS database, also the fragmentation pathways are in agreement and well discussed in the literature [94].

Compounds 9 and 12 were characterized as chromone derivatives. However, as far as we know, studies via mass spectrometry for chromones have rarely been performed in electrospray ionization in negative mode. It is still difficult to propose coherent fragmentation pathways and mechanisms with fragment substructures. Based on several studies on fragmentation reactions [42,95–111] in low molecular weight natural products, we tentatively proposed some loss neutral from the fragmentation of these compounds.

Compound 9 has been reported in the literature as aloesol [M–H]⁻ of *m/z* 233.0810. Its main fragment of *m/z* 189 [(M-H)-CO_2]⁻ is produced through the loss of carbon dioxide [49]. The compound 12 clusterized directly with 9 in the molecular network with a cosine score 0.93, suggesting a close structure relation. It was identified as 7-hydroxy-2,5-dimethyl-4H-chromen- 4-one with [M-H]⁻ of *m/z* 189.0545. It showed a main fragment pf *m/z* 174 [(M-H)-CH_3]$^{-\bullet}$ corresponding to the loss of the methyl radical from position C-2 or C-3.

1.2. Flavones, Flavanonol and Flavanone Derivatives

Compound 17 detected as [M–H]⁻ of *m/z* 285.0396 was putatively identified as luteolin, a molecule reported already in the genus *Chamaecrista* [30,112]. The fragmentation pattern presented ions of *m/z* 241[(M-H)-CO_2]⁻ produced through rearrangement of the ring C by loss of CO_2 . The fragment of *m/z* 217 was likely produced through the cleavage of ring A [(M-H)-C_3O_2]⁻, involving an unusual hydrogen rearrangement [113–115]. The *m/z* 133 [(M-H)-$C_7H_4O_4$]⁻ was generated through a $^{1,3}B^-$ fragmentation mechanism [116] and the loss of CO_2.

Compound 8 was characterized as luteolin-7-*O*-glucoside and exhibited [M-H]⁻ *m/z* at 447.0925. Unlike isomers 2, 3 and 4, compound 8 contains a glycoside at position C-7, as suggested by the intensity of the aglycone base peak at *m/z* 285 [(M-H)-$C_6H_{10}O_5$]⁻, which is characteristic of a O-glycosidic bond in the flavonoid family. This compound has previously been reported in the genus with an hexose [117]. The ions at *m/z* 357 and *m/z* 339, correspond to a glycoside fragmentation type [(M-H)-$C_3H_6O_3$]⁻ ($^{0,3}X_1$) and a dehydration [(M-H)-$C_3H_6O_3$-H_2O]⁻ ($^{0,2}X_1$), respectively. The loss of methanal [(M-H)-$C_3H_6O_3$-CH_2O]⁻ is observed between ions at *m/z* 339 and at *m/z* 327 [118–120], followed by the loss of two carbon monoxide at *m/z* 311 and *m/z* 299 [(M-H)-$C_3H_6O_3$-H_2O-2CO]⁻. At *m/z* 285 is aglycone and the fragment *m/z* 255 [(M-H)-$C_6H_{10}O_5$-CH_2O]⁻ occurs with a loss of methanal. The ion *m/z* 151 [(M-H)-$C_6H_{10}O_5$-$C_8H_6O_2$]⁻ ($^{1,3}A^-$) is produced by the cleavage of ring C of the aglycone at *m/z* 133 [(M-H)-$C_6H_{10}O_5$-$C_7H_4O_4$]⁻ ($^{1,3}B^-$) through Retro–Diels–Alder (RDA).

Compound 10 is a flavanonol already reported in the literature [121] and the fragmentation pattern has been confirmed in GNPS. It was characterized as dihydrokaempferol [M-H]⁻ *m/z* at 287.0551 and its main fragments were at *m/z* 269 for dehydration on the C-3 position, *m/z* 259 for the loss of CO on the C ring, *m/z* 243 for the loss of CO_2 also in the C ring following at *m/z* 201 for the loss of C_2H_2O on the B ring, and *m/z* 151 by RDA (mechanism $^{1,3}A^-$). The compound 14, detected at m/z 461.1072, was characterized as 5,7-dihydroxy-6-methoxy-2-[4-[(2S,3R,4S,5S,6R)-3,4,5-trihydroxy-6-(hydroxymethyl)oxan-2-yl]oxyphenyl]chromen-4-one and showed fragments, confirmed on GNPS, corresponding to the heterolytic cleavage and loss of CH_3 at *m/z* 446 and the loss of an hexose at *m/z* 299.

Peak 22 [M-H]⁻ at *m/z* 271.0601 was putatively characterized as naringenin. It presented fragments at *m/z* 253 [(M–H)–H_2O]⁻, a dehydration, and *m/z* 227 [(M-H)-CO_2]⁻ loss of neutral carbon dioxide followed by *m/z* 199 [(M-H)-CO_2-CO]⁻ corresponding to the further loss of a carbon monoxide [122]. The ions *m/z* 177 [(M-H)-C_6H_6O]⁻ and 151 [(M-H)-C_8H_8O]⁻ are well discussed in the literature [123]. Compound 23, also reported in the genus [30], was identified as apigenin with [M-H]⁻ at *m/z* 269.0447 and fragments at *m/z* 225 [(M-H)-CO_2]⁻ and *m/z* 151 [(M-H)-C_8H_6O]⁻.

1.3. Isocoumarin

Peak 13 is an isocoumarin detected [M-H]⁻ at *m/z* 191.0360 and confirmed with standard on the database. This was characterized as 1H-2-benzopyran-1-one,6,8-dihydroxy-3-methyl and produced fragments at *m/z* 176 [(M-H)-CH_3]⁻ and *m/z* 149 [(M-H)-C_2H_2O]⁻.

1.4. Carboxylic Acid Derivatives

The carboxylic acids detected in this study are already known in natural plant products, as well as their fragmentation patterns (spectrum ID on Table 1). The peak 11 [M–H]⁻ at *m/z* 187.0965 corresponds to azelaic acid, (peak 27 [M-H]⁻ at *m/z* 327.2165), 9,12,13-

Trihydroxy-10(*E*),15(*Z*)-octadecadienoic acid (peaks 28 and 30 [M-H]⁻ at *m/z* 227.12 as butanedioic acid,2-(4,4-dimethyl-2-methylenepentyl) isomers), peak 31 [M-H]⁻ at *m/z* 329.2320 as 9,12,13-Trihydroxyoctadec-10-enoic acid and peak 35 [M-H]⁻ at *m/z* 293.2115 as hydroxyoctadecatrienoic acid.

1.5. Naphthalene Derivatives

Compounds 15, 16 and 18 were characterized as naphthalene derivatives, torachrysone-8-hexosyl-hexoside ([M-H]⁻ at *m/z* 569.1868) and two torachrysone-8-hexosyl-pentoside isomers ([M-H]⁻ at *m/z* 539.1760 and *m/z* 539.1770), respectively. The presence of this type of compound is unusual, even though they have been reported in species from the genus *Cassia* [123–126] from which the genus *Chamaecrista* was segregated [30]. Fragmentation patterns are similar between them. For compound 15, the base peak at *m/z* 245 [(M-H)-$C_{12}H_{20}O_{10}$]⁻ is produced through the loss of the disaccharide in the C-8 position [116,127], which possibly corresponds to a hexosyl-hexoside. The same happened for compound 16 and 18, *m/z* 245 [(M-H)-$C_{11}H_{18}O_9$]⁻, but the disaccharide possibly corresponds to a hexosyl-pentoside, such as disaccharide type xylosyl-(1→2)-glucose) [128]. Other fragments were observed for all, as at *m/z* 230 [(M-H)-CH_3]⁻• and *m/z* 215 [(M-H)-CH_3-CH_3]⁻• were produced through two homolytic cleavages causing the loss of a methyl radical [126].

1.6. Anthraquinones and Xanthone Derivatives

Peak 32 was identified as norlichexanthone [M-H]⁻ at *m/z* 257.0442 and. This was confirmed on a data bank and showed a fragmentation pattern with a base peak at *m/z* 213 [(M-H)-CO_2]⁻ (a neutral loss of a carbon dioxide from ring B). The peak 34 detected [M-H]⁻ at *m/z* 257.0444 was characterized as emodin and the fragmentation was initiated by the elimination of CO to produce *m/z* 241 [(M-H)-CO]⁻ [129], and the loss of a CO_2 to give *m/z* 225 [(M-H)-CO_2]⁻ [130].

Compound 24 and 33 were characterized as two carviolin isomers [M-H]⁻ at *m/z* 299.0554 and *m/z* 299.0550, respectively. The main fragments observed were at *m/z* 284 [(M-H)-CH_3]⁻•, produced by an homolytic cleavage of the methoxyl present in C-4' of the ring B and *m/z* 256 [(M-H)-CH_3-CO_2]⁻ corresponds to the loss of carbon dioxide. The data on fragmentation patterns have confirmed this compound in GNPS.

References

30. Quirós-Guerrero, L.; Albertazzi, F.; Araya-Valverde, E.; Romero, R.M.; Villalobos, H.; Poveda, L.; Chavarría, M.; Tamayo-Castillo, G. Phenolic variation among Chamaecrista nictitans subspecies and varieties revealed through UPLC-ESI(-)-MS/MS chemical fingerprinting. *Metabolomics* **2019**, *15*, 14, doi:10.1007/s11306-019-1475-8.
31. Mateos-Martín, M.L.; Fuguet, E.; Jiménez-Ardón, A.; Herrero-Uribe, L.; Tamayo-Castillo, G.; Torres, J.L. Identification of polyphenols from antiviral Chamaecrista nictitans extract using high-resolution LC-ESI-MS/MS. *Anal. Bioanal. Chem.* **2014**, *406*, 5501–5506, doi:10.1007/s00216-014-7982-6.
32. Tangavelou, A.C.; Viswanathan, M.B.; Balakrishna, K.; Patra, A. Phytochemical Analysis in the Leaves of Chamaecrista nigricans (Leguminosae). *Pharm. Anal. Acta.* **2018**, *9*, 582. doi: 10.4172/2153-2435.1000582
33. Sebei, K.; Sbissi, I.; Souhir, A.; Herchi, W.; Ssakouhi, F.; Boukhchina, S. Phylogenetic identification, phytochemical analysis and antioxidant activity of Chamaecrista absus var. absus seeds. *J. Plant. Biol. Res.* **2014**, *3*, 1–11.
34. Barba, B.; G. Díaz, J.; Herz, W. Cassanes and anthraquinones from Chamaecrista greggii. *Phytochemistry* **1994**, *37*, 837–845, doi:10.1016/S0031-9422(00)90367-4.
42. Ma, Y.L.; Vedernikova, I.; Van den Heuvel, H.; Claeys, M. Internal glucose residue loss in protonated O-diglycosyl flavonoids upon low-energy collision-induced dissociation. *J. Am. Soc. Mass Spectrom.* **2000**, *11*, 136–144, doi:10.1016/S1044-0305(99)00133-6.
49. Azizah, M.; Pripdeevech, P.; Thongkongkaew, T.; Mahidol, C.; Ruchirawat, S.; Kittakoop, P. UHPLC-ESI-QTOF-MS/MS-Based Molecular Networking Guided Isolation and Dereplication of Antibacterial and Antifungal Constituents of Ventilago denticulata. *Antibiotics* **2020**, *9*, doi:10.3390/antibiotics9090606.
89. Barba, B.; Diaz, J.G.; Goedken, V.L.; Herz, W.; Dominguez, X.A. Unusual cassanes from a Chamaecrista species. *Tetrahedron* **1992**, *48*, 4725–4732, doi:10.1016/s0040-4020(01)81569-6.
90. Crockett, S.L.; Kunert, O.; Pferschy-Wenzig, E.-M.; Jacob, M.; Schuehly, W.; Bauer, R. Phloroglucinol and Terpenoid Derivatives from Hypericum cistifolium and H. galioides (Hypericaceae). *Front. Plant Sci.* **2016**, *7*, 961, doi:10.3389/fpls.2016.00961.
91. Dewick, P.M. *Medicinal Natural Products: A Biosynthetic Approach*; John Wiley & Sons, 2011; ISBN 9781119964575.

92. Chosson, E.; Chaboud, A.; Chulia, A.J.; Raynaud, J. A phloracetophenone glucoside from Rhododendron ferrugineum. *Phytochemistry* **1998**, *47*, 87–88, doi:10.1016/S0031-9422(97)00475-5.
93. Engels, C.; Schieber, A.; Gänzle, M.G. Sinapic acid derivatives in defatted Oriental mustard (Brassica juncea L.) seed meal extracts using UHPLC-DAD-ESI-MS n and identification of compounds with antibacterial activity. *Eur. Food Res. Technol.* **2012**, *234*, 535–542, doi:10.1007/s00217-012-1669-z.
94. Oliveira, D.N.; Ferreira, M.S.; Catharino, R.R. Rapid and simultaneous in situ assessment of aflatoxins and stilbenes using silica plate imprinting mass spectrometry imaging. *PLoS One* **2014**, *9*, e90901, doi:10.1371/journal.pone.0090901.
95. Justesen, U. Collision-induced fragmentation of deprotonated methoxylated flavonoids, obtained by electrospray ionization mass spectrometry. *J. Mass Spectrom.* **2001**, *36*, 169–178, doi:10.1002/jms.118.
96. Xia, B.; Li, J.; Mei, W.; Ding, L.; Xu, H.; Zhou, Y. Tandem mass spectrometry fragmentation of the protonated 2-(2-phenylethyl)chromones from agarwood: radical ions versus non-radical ions. *J. Mass Spectrom.* **2013**, *48*, 979–982, doi:10.1002/jms.3242.
97. Su, S.; Wang, Y.; Bai, L.; Xia, B.; Li, X.; Tang, Y.; Xu, P.; Xue, M. Structural elucidation of in vivo metabolites of isobavachalcone in rat by LC-ESI-MS(n) and LC-NMR. *J. Pharm. Biomed. Anal.* **2015**, *104*, 38–46, doi:10.1016/j.jpba.2014.11.010.
98. Li, H.-J.; Deinzer, M.L. Tandem Mass Spectrometry for Sequencing Proanthocyanidins. *Anal. Chem.* 2007, *79*, 1739–1748.
99. Friedrich, W.; Eberhardt, A.; Galensa, R. Investigation of proanthocyanidins by HPLC with electrospray ionization mass spectrometry. *Eur. Food Res. and Technol.* 2000, *211*, 56–64.
100. Jaiswal, R.; Jayasinghe, L.; Kuhnert, N. Identification and characterization of proanthocyanidins of 16 members of the Rhododendron genus (Ericaceae) by tandem LC-MS. *J. Mass Spectrom.* **2012**, *47*, 502–515, doi:10.1002/jms.2954.
101. Reis, A.; Domingues, P.; Ferrer-Correia, A.J.V.; Domingues, M.R.M. Tandem mass spectrometry of intact oxidation products of diacylphosphatidylcholines: evidence for the occurrence of the oxidation of the phosphocholine head and differentiation of isomers. *J. Mass Spectrom.* **2004**, *39*, 1513–1522, doi:10.1002/jms.751.
102. 1Ponomarev, D.; Takhistov, V. Thermochemistry of Organic and Heteroorganic Species. Part 19. Structural Aspects and Thermochemical Approach to Isomerization and Fragmentation of Negative Ions: Skeletal Rearrangements, Molecular Ion and Artifacts. *ChemInform* 2006, *37*.
103. Griffiths, W.J.; Yang, Y.; Sjövall, J.; Lindgren, J.A. Electrospray-collision-induced dissociation mass spectrometry of mono-, di- and tri-hydroxylated lipoxygenase products, including leukotrienes of the B-series and lipoxins. *Rapid Commun. Mass Spectrom.* **1996**, *10*, 183–196, doi:3.0.CO;2-W">10.1002/(SICI)1097-0231(19960131)10:2<183::AID-RCM456>3.0.CO;2-W.
104. Crevelin, E.J.; Crotti, A.E.M.; Zucchi, T.D.; Melo, I.S.; Moraes, L.A.B. Dereplication ofStreptomycessp. AMC 23 polyether ionophore antibiotics by accurate-mass electrospray tandem mass spectrometry. *J. Mass Spectrometry* 2014, *49*, 1117–1126.
105. Fredenhagen, A.; Derrien, C.; Gassmann, E. An MS/MS library on an ion-trap instrument for efficient dereplication of natural products. Different fragmentation patterns for [M + H]+ and [M + Na]+ ions. *J. Nat. Prod.* **2005**, *68*, 385–391, doi:10.1021/np049657e.
106. Lopes, N.P.; Gates, P.J.; Wilkins, J.P.G.; Staunton, J. Fragmentation studies on lasalocid acid by accurate mass electrospray mass spectrometry. *The Analyst* 2002, *127*, 1224–1227.
107. Crotti, A.E.M.; Lopes, J.L.C.; Lopes, N.P. Triple quadrupole tandem mass spectrometry of sesquiterpene lactones: a study of goyazensolide and its congeners. *J. Mass Spectrom.* **2005**, *40*, 1030–1034, doi:10.1002/jms.877.
108. Wang, H.; Wu, Y.; Zhao, Z. Fragmentation study of simvastatin and lovastatin using electrospray ionization tandem mass spectrometry. *J. Mass Spectrom.* **2001**, *36*, 58–70, doi:10.1002/jms.104.
109. Hong, H.; Gates, P.J.; Staunton, J.; Stinear, T.; Cole, S.T.; Leadlay, P.F.; Spencer, J.B. Identification using LC-MSn of co-metabolites in the biosynthesis of the polyketide toxin mycolactone by a clinical isolate of Mycobacterium ulcerans. *Chem. Commun.* **2003**, 2822–2823, doi:10.1039/b308163j.
110. Kleigrewe, K.; Aydin, F.; Hogrefe, K.; Piecuch, P.; Bergander, K.; Würthwein, E.-U.; Humpf, H.-U. Structure elucidation of new fusarins revealing insights in the rearrangement mechanisms of the Fusarium mycotoxin fusarin C. *J. Agric. Food Chem.* **2012**, *60*, 5497–5505, doi:10.1021/jf3009469.
111. Irwin, H.S.; Barneby, R.C. *The american Cassiinae : a synoptical revision of Leguminosae tribe Cassieae subtribe Cassiinae in the new world. 1 (1982)*; Scientific Publications Department, 1982;.
112. Troalen, L.G.; Phillips, A.S.; Peggie, D.A.; Barran, P.E.; Hulme, A.N. Historical textile dyeing with Genista tinctoria L.: a comprehensive study by UPLC-MS/MS analysis. *Anal. Methods* 2014, *6*, 8915–8923.
113. Schmidt, J. Negative ion electrospray high-resolution tandem mass spectrometry of polyphenols. *J. Mass Spectrom.* **2016**, *51*, 33–43, doi:10.1002/jms.3712.
114. Desta, K.T.; Kim, G.S.; Abd El-Aty, A.M.; Raha, S.; Kim, M.-B.; Jeong, J.H.; Warda, M.; Hacımüftüoğlu, A.; Shin, H.-C.; Shim, J.-H.; et al. Flavone polyphenols dominate in Thymus schimperi Ronniger : LC–ESI–MS/MS characterization and study of anti-proliferative effects of plant extract on AGS and HepG2 cancer cells. *J. Chromatogr. B* 2017, *1053*, 1–8.
115. Ma, Y.L.; Li, Q.M.; Van den Heuvel, H.; Claeys, M. Characterization of flavone and flavonol aglycones by collision-induced dissociation tandem mass spectrometry. *Rapid Commun. in Mass Spectrom.* 1997, *11*, 1357–1364.
116. Frański, R.; Matławska, I.; Bylka, W.; Sikorska, M.; Fiedorow, P.; Stobiecki, M. Differentiation of interglycosidic linkages in permethylated flavonoid glycosides from linked-scan mass spectra (B/E). *J. Agric. Food Chem.* **2002**, *50*, 976–982, doi:10.1021/jf011082t.
117. March, R.E.; Miao, X.-S. A fragmentation study of kaempferol using electrospray quadrupole time-of-flight mass spectrometry at high mass resolution. *Int. J. Mass Spectrom.* **2004**, *231*, 157–167, doi:10.1016/j.ijms.2003.10.008.
118. Hughes, R.J.; Croley, T.R.; Metcalfe, C.D.; March, R.E. A tandem mass spectrometric study of selected characteristic flavonoids.

Int. J. Mass Spectrom. **2001**, *210-211*, 371–385, doi:10.1016/s1387-3806(01)00451-1.
119. Sánchez-Rabaneda, F.; Jáuregui, O.; Casals, I.; Andrés-Lacueva, C.; Izquierdo-Pulido, M.; Lamuela-Raventós, R.M. Liquid chromatographic/electrospray ionization tandem mass spectrometric study of the phenolic composition of cocoa (Theobroma cacao). *J. Mass Spectrom.* **2003**, *38*, 35–42, doi:10.1002/jms.395.
120. Wu, W.; Yan, C.; Li, L.; Liu, Z.; Liu, S. Studies on the flavones using liquid chromatography–electrospray ionization tandem mass spectrometry. *J. Chromatogr.* **2004**, *1047*, 213–220.
121. Chen, G.; Li, X.; Saleri, F.; Guo, M. Analysis of Flavonoids in Rhamnus davurica and Its Antiproliferative Activities. *Molecules* **2016**, *21*, doi:10.3390/molecules21101275.
122. Xu, F.; Liu, Y.; Zhang, Z.; Yang, C.; Tian, Y. Quasi-MSn identification of flavanone 7-glycoside isomers in Da Chengqi Tang by high performance liquid chromatography-tandem mass spectrometry. *Chin. Med.* **2009**, *4*, 15, doi:10.1186/1749-8546-4-15.
123. Zeng, X.; Su, W.; Zheng, Y.; Liu, H.; Li, P.; Zhang, W.; Liang, Y.; Bai, Y.; Peng, W.; Yao, H. UFLC-Q-TOF-MS/MS-Based Screening and Identification of Flavonoids and Derived Metabolites in Human Urine after Oral Administration of Exocarpium Citri Grandis Extract. *Molecules* **2018**, *23*, doi:10.3390/molecules23040895.
124. Dong, X.; Fu, J.; Yin, X.; Yang, C.; Zhang, X.; Wang, W.; Du, X.; Wang, Q.; Ni, J. Cassiae semen: A review of its phytochemistry and pharmacology (Review). *Mol. Med. Rep.* **2017**, *16*, 2331–2346, doi:10.3892/mmr.2017.6880.
125. El-Halawany, A.M.; Chung, M.H.; Nakamura, N.; Ma, C.-M.; Nishihara, T.; Hattori, M. Estrogenic and anti-estrogenic activities of Cassia tora phenolic constituents. *Chem. Pharm. Bull.* **2007**, *55*, 1476–1482, doi:10.1248/cpb.55.1476.
126. Hatano, T.; Uebayashi, H.; Ito, H.; Shiota, S.; Tsuchiya, T.; Yoshida, T. Phenolic constituents of Cassia seeds and antibacterial effect of some naphthalenes and anthraquinones on methicillin-resistant Staphylococcus aureus. *Chem. Pharm. Bull.* **1999**, *47*, 1121–1127, doi:10.1248/cpb.47.1121.
127. Wang, M.; Fu, J.; Guo, H.; Tian, Y.; Xu, F.; Song, R.; Zhang, Z. Discrimination of crude and processed rhubarb products using a chemometric approach based on ultra fast liquid chromatography with ion trap/time-of-flight mass spectrometry. *J. Sep. Sci.* 2015, *38*, 395–401.
128. Saito, N.; Tatsuzawa, F.; Nishiyama, A.; Yokoi, M.; Shigihara, A.; Honda, T. Acylated cyanidin 3-sambubioside-5-glucosides in Matthiola incana. *Phytochemistry* **1995**, *38*, 1027–1032, doi:10.1016/0031-9422(94)00659-h.
129. Ye, M.; Han, J.; Chen, H.; Zheng, J.; Guo, D. Analysis of phenolic compounds in rhubarbs using liquid chromatography coupled with electrospray ionization mass spectrometry. *J. Am. Soc. Mass Spectrom.* **2007**, *18*, 82–91, doi:10.1016/j.jasms.2006.08.009.
130. Shahid, M.; Wertz, J.; Degano, I.; Aceto, M.; Khan, M.I.; Quye, A. Analytical methods for determination of anthraquinone dyes in historical textiles: A review. *Anal. Chim. Acta* **2019**, *1083*, 58–87, doi:10.1016/j.aca.2019.07.009.
